# *Spic* regulates one-carbon metabolism and histone methylation in ground-state pluripotency

**DOI:** 10.1126/sciadv.adg7997

**Published:** 2023-08-18

**Authors:** Fatemeh Mirzadeh Azad, Eduard A. Struys, Victoria Wingert, Luciana Hannibal, Ken Mills, Joop H. Jansen, Daniel B. Longley, Hendrik G. Stunnenberg, Yaser Atlasi

**Affiliations:** ^1^ Patrick G Johnston Centre for Cancer Research, Queen’s University Belfast, Belfast, UK.; ^2^ Department of Clinical Chemistry, Amsterdam University Medical Center, Amsterdam, Netherlands.; ^3^ Laboratory of Clinical Biochemistry and Metabolism, Department of General Pediatrics, Adolescent Medicine and Neonatology, Faculty of Medicine, Medical Center, University of Freiburg, Freiburg, Germany.; ^4^ Department of Laboratory Medicine, Radboud University Nijmegen Medical Centre, Nijmegen, Netherlands.; ^5^ Department of Molecular Biology, Faculty of Science, Radboud University, Nijmegen, Netherlands.; ^6^ Princess Maxima Centre for Pediatric Oncology, Utrecht, Netherlands.

## Abstract

Understanding mechanisms of epigenetic regulation in embryonic stem cells (ESCs) is of fundamental importance for stem cell and developmental biology. Here, we identify *Spic*, a member of the ETS family of transcription factors (TFs), as a marker of ground state pluripotency. We show that *Spic* is rapidly induced in ground state ESCs and in response to extracellular signal–regulated kinase (ERK) inhibition. We find that SPIC binds to enhancer elements and stabilizes NANOG binding to chromatin, particularly at genes involved in choline/one-carbon (1C) metabolism such as *Bhmt*, *Bhmt2*, and *Dmgdh*. Gain-of-function and loss-of-function experiments revealed that *Spic* controls 1C metabolism and the flux of *S*-adenosyl methionine to *S*-adenosyl-L-homocysteine (SAM-to-SAH), thereby, modulating the levels of H3R17me2 and H3K4me3 histone marks in ESCs. Our findings highlight betaine-dependent 1C metabolism as a hallmark of ground state pluripotency primarily activated by SPIC. These findings underscore the role of uncharacterized auxiliary TFs in linking cellular metabolism to epigenetic regulation in ESCs.

## Teaser

Spic regulates one carbon metabolism and epigenetic modification in ground state pluripotency.

## INTRODUCTION

During early embryonic development, cells transit through a series of pluripotent states, some of which have been captured in vitro under specific culture conditions as embryonic stem cell (ESCs). For example, mouse ESCs (mESCs) cultured with leukemia inhibitory factor (LIF), as well as inhibitors of glycogen synthase kinase 3 (GSK3) and mitogen-activated protein kinase kinase (MEK) (2iL-ESCs), closely represent the ground-state pluripotency found in the preimplantation embryo, whereas ESCs cultured with fibroblast growth factor 2 (FGF2) and Activin (EPI) resemble the postimplantation epiblast. ESCs can also be cultured with serum and LIF (SL-ESCs) that represent a metastable state with features of both preimplantation and postimplantation epiblasts (*1*, *2*). These ESC states represent snapshots of the pluripotency spectrum and provide a versatile model for understanding the molecular basis of pluripotency.

Transition between different states of pluripotency is associated with global transcriptional and epigenetic reprogramming that restricts the developmental potential of stem cells, allowing them to undergo lineage commitment (*1*). However, the functions of many 2iL-specific transcription factors (TFs) remain enigmatic, partly because of the resilience of the cells to epigenetic perturbations, highlighting a gap in our knowledge of additional mechanisms that stabilize the epigenetic landscape in ground-state pluripotency. To identify new pluripotency-associated markers, we have recently examined the temporal transcriptional and epigenetic alterations between different states of ESCs and noted *Spic* as one of the earliest induced genes in 2iL-ESCs (*3*). In addition, a recent study has performed an interspecies transcriptional comparison of pluripotent cells and highlighted *Spic* as a conserved marker of inner cell mass (ICM) (*4*). *Spic* is a member of the E26 transformation–specific (ETS) family of TFs with high similarity to *Spib* and *Spi1* (PU.1) (*5*, *6*). *Spic* has been studied in the context of the immune system where it is expressed in splenic red pulp macrophages (RPM) (*6*–*9*), in B cells (*5*, *10*–*12*), and at a lower level in gut macrophages (*13*, *14*). In immune cells, *Spic* instructs differentiation of monocytes to RPM (*8*, *9*), as well as B cells to plasma cells (*12*). However, the role of *Spic* in ESCs and early embryonic development remains unknown. Here, we show that *Spic* functions as an auxiliary TF in chromatin regulation and plays a key role in one-carbon (1C) metabolism to control the level of *S*-adenosyl methionine (SAM) and histone methylation in ground-state pluripotency.

## RESULTS

### *Spic* expression marks ground-state pluripotency

**Fig. 1.**
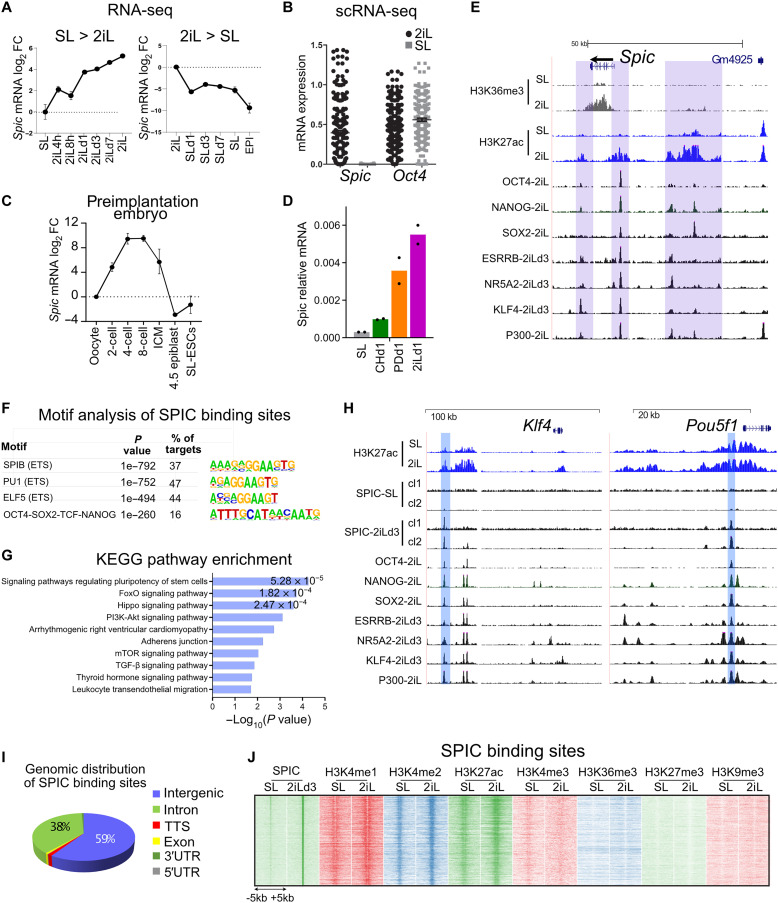
*Spic* is a specific marker of ground-state pluripotency. (**A**) RNA-seq showing that *Spic* is highly induced in 2iL-ESCs. The dots show the mean, and the error bars show the SEM of *n* = 2 biological replicates. (**B**) Single-cell RNA-seq (scRNA-seq) showing *Spic* expression in 2iL-ESCs but not SL-ESCs (*n* = 190 cells). *Oct4* expression is shown as the control. Each dot represents one cell. (**C**) RNA-seq showing *Spic* expression during the preimplantation stages of mouse embryo. The dots show the mean, and the error bars show the SEM of *n* > 2 biological replicates. (**D**) Quantitative reverse transcription polymerase chain reaction (qRT-PCR) showing *Spic* induction downstream of PD0325901 treatment (MEK/ERK inhibition). The bars show the mean of *n* = 2 biological replicates. (**E**) Genome browser view showing that the *Spic* locus is occupied by different pluripotency factors. (**F**) Motif analysis of SPIC chromatin binding sites showing enrichment for ETS and OCT4/SOX2/TCF/NANOG motifs. (**G**) Gene ontology term enrichment analysis for genes located in the vicinity of SPIC binding sites. (**H**) Genome browser view showing two representative examples of SPIC target genes. (**I**) Genome-wide distribution of SPIC binding sites in 2iL-ESCs. (**J**) SPIC binding sites are demarked by histone modifications associated with enhancers. FC, fold change; KEGG, Kyoto Encyclopedia of Genes and Genomes; PI3K, phosphatidylinositol 3-kinase; mTOR, mammalian target of rapamycin; TGF-β, transforming growth factor–β; TTS, transcription termination site; 3′UTR, 3′ untranslated region; 5′UTR, 5′ untranslated region.

We analyzed the transcriptional profiles of mESCs maintained in different states of pluripotency (*3*, *15*) and detected a high level of *Spic* in 2iL-ESCs that is rapidly down-regulated in SL-ESCs (by ~32-fold) and is fully repressed in EPI-ESCs (Fig. 1A). Single-cell RNA sequencing (RNA-seq) analysis of SL- and 2iL-ESCs (*16*) further confirmed that *Spic* is not expressed in SL-ESCs (Fig. 1B). RNA-seq of mouse preimplantation embryos (*17*) demonstrated that *Spic* is highly expressed in four-cell, eight-cell, and ICM embryonic stages but is sharply down-regulated in postimplantation epiblasts (Fig. 1C). As 2iL culture is based on two kinase inhibitors (CHIR99021 and PD0325901), we next cultured ESCs in media supplemented with the single inhibitors and found that *Spic* is mainly induced by PD0325901 treatment, suggesting that *Spic* is negatively regulated by MEK/extracellular signal–regulated kinase (ERK) signaling (Fig. 1D). Consistently, analysis of published chromatin immunoprecipitation sequencing (ChIP-seq) data (*18*) confirmed ERK binding at *Spic* promoter in SL-ESCs, which was diminished in 2iL- and PD0325901-treated ESCs (fig. S1A). In line with its expression in 2iL-ESCs, the *Spic* locus is occupied by pluripotency-associated TFs, including OCT4 (Octamer-binding transcription factor 4), SOX2 (SRY-box transcription factor 2), NANOG, ESRRB (Estrogen related receptor beta), KLF4 (Kruppel like factor 4), and NR5A2 (Nuclear receptor subfamily 5 group A member 2) (Fig. 1E).

We next investigated the genome-wide binding of SPIC in 2iL-ESCs. Using ESCs expressing bacterial artificial chromosomes (BACs) containing SPIC–green fluorescent protein (GFP) fusion protein, we performed GFP–ChIP-seq in ESCs cultured in SL or 3 days of SL-to-2iL transition (2iLd3) and detected 2379 high-confidence SPIC-GFP peaks in two independent ESC clones. These peaks represent early SPIC binding sites and are maintained in long-term 2iL-cultured ESCs (2iLd25; fig. S1, B and C). Motif analysis of SPIC ChIP-seq peaks showed high enrichment for the ETS motifs, confirming the specificity of our ChIP-seq data (Fig. 1F). We also detected high enrichment of OCT4-NANOG-SOX2-TCF3 motif at SPIC binding sites, indicating co-occupancy by the core pluripotency factors. Analysis of ChIP-seq data collected in 2iL-ESCs (*19*) further confirmed that ~70% of SPIC binding sites overlap with OCT4, SOX2, or NANOG binding (fig. S1D). Gene ontology term enrichment analysis confirmed that SPIC binding sites are located at the vicinity of genes involved in pluripotency (Fig. 1, G and H). We noted that most SPIC peaks are located at intergenic (~60%) or intronic regions (38%; Fig. 1I), which are characterized by high levels of H3K4me1/2 and H3K27ac enhancer marks, low levels of H3K4me3, and the absence of H3K9me3 and H3K27me3 repressive marks (Fig. 1J). Collectively, these data demonstrate that SPIC is a specific marker of ground-state pluripotency that binds distal regulatory elements of active genes associated with ESC pluripotency.

### SPIC stabilizes NANOG binding at genes involved in 1C metabolism

**Fig. 2.**
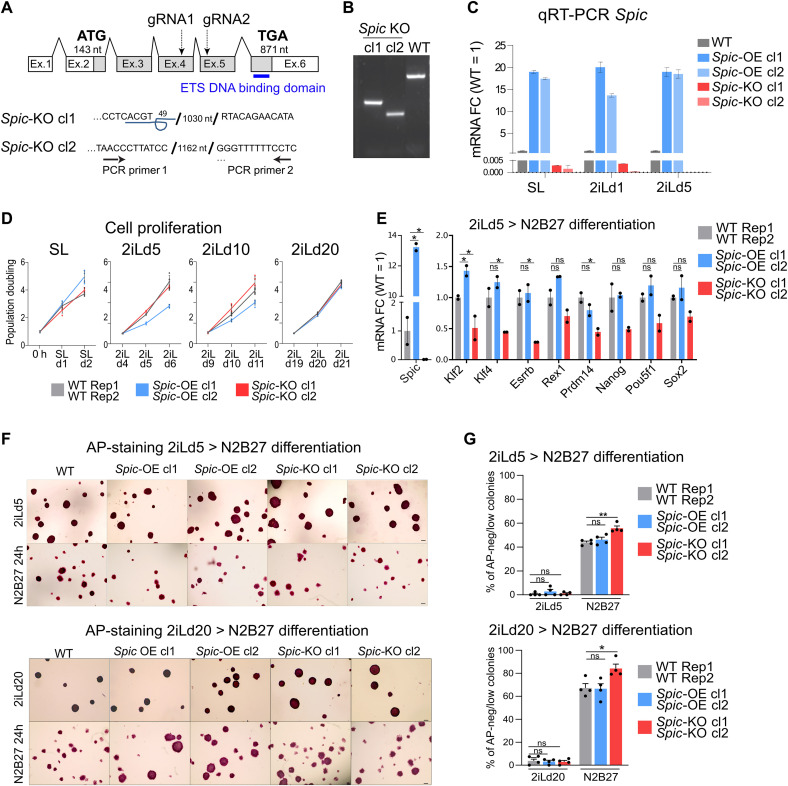
SPIC supports 2iL-ESCs but is not essential for ESC maintenance. (**A**) Schematic representation of *Spic* locus and locations of guide RNA (gRNA)/PCR primers used for generating *Spic*-KO ESCs. The sequencing results obtained in two independent *Spic*-KO clones are shown at the junction of the deleted DNA fragments. (**B**) PCR validation of *Spic*-KO ESCs using genomic DNA. (**C**) qRT-PCR analysis showing *Spic* expression in *Spic*-WT, *Spic*-KO, and *Spic*-OE cells. Two independent clones for *Spic*-KO and *Spic*-OE were used. The bars represent the means, and the error bars show the SEM of *n* = 2 technical replicates. (**D**) Graph showing the proliferation rate of *Spic*-KO, *Spic*-OE, and *Spic*-WT ESCs in SL or at different time points of SL-to-2iL transition. The lines represent the mean of two independent clones and *n* = 3 biological replicates per clone for *Spic*-KO and *Spic*-OE ESCs and *n* = 6 biological replicates for WT ESCs. (**E**) qRT-PCR showing expression of pluripotency markers during early differentiation (24 hours) of ESCs in N2B27 medium. The bars represent the means of *n* = 2 independent clones of *Spic*-KO or *Spic*-OE and *n* = 2 biological replicates of WT ESCs. (**F**) Microscopy pictures showing the AP staining in *Spic*-WT, *Spic*-KO, and *Spic*-OE ESCs cultured in 2iL (day 5 or day 20) or differentiated for 24 hours in N2B27 medium. Scale bars, 200 μm. (**G**) Graphs showing the percentage of AP-low colonies upon N2B27 differentiation in different ESCs. The bars represent the means of two independent clones and *n* = 2 biological replicates per clone for *Spic*-KO or *Spic*-OE, and *n* = 4 biological replicates for WT ESCs. In all graphs, the error bars show the SEM and the asterisks represent *P* < 0.05, unpaired two-tailed Student’s *t* test. ns, not significant.

To study the role of *Spic* in ground-state pluripotency, we generated *Spic*–knockout (KO) ESCs using CRISPR-Cas9 and *Spic*–overexpressing (OE) ESCs using SPIC-GFP BACs and selected two independent ESC clones per genotype for downstream analysis (Fig. 2, A to C, and fig. S1E). We observed that both *Spic*-KO and *Spic*-OE ESCs maintained normal morphology, proliferation rate, and expression of key pluripotency markers in SL and 2iL states, suggesting that *Spic* is not indispensable for ESC maintenance (Fig. 2D and fig. S1, F to H). However, we noted that *Spic* attenuates the early exit from ground-state pluripotency. Differentiation of ESCs in N2B27 medium showed that *Spic*-KO cells exhibit accelerated differentiation capacity as measured by reduced levels of naïve pluripotency markers and the number of alkaline phosphatase (AP)–positive colonies when compared to *Spic*-OE and wild-type (WT) control ESCs (Fig. 2, E to G). Similarly, resetting cells from epiblast-like stem cells (EpiLSCs) to 2iL showed that *Spic* can enhance the EpiLSC-to-2iL resetting efficiency (fig. S2A). Thus, *Spic* supports cells in 2iL state but is not essential for maintenance of ground-state pluripotency.

**Fig. 3.**
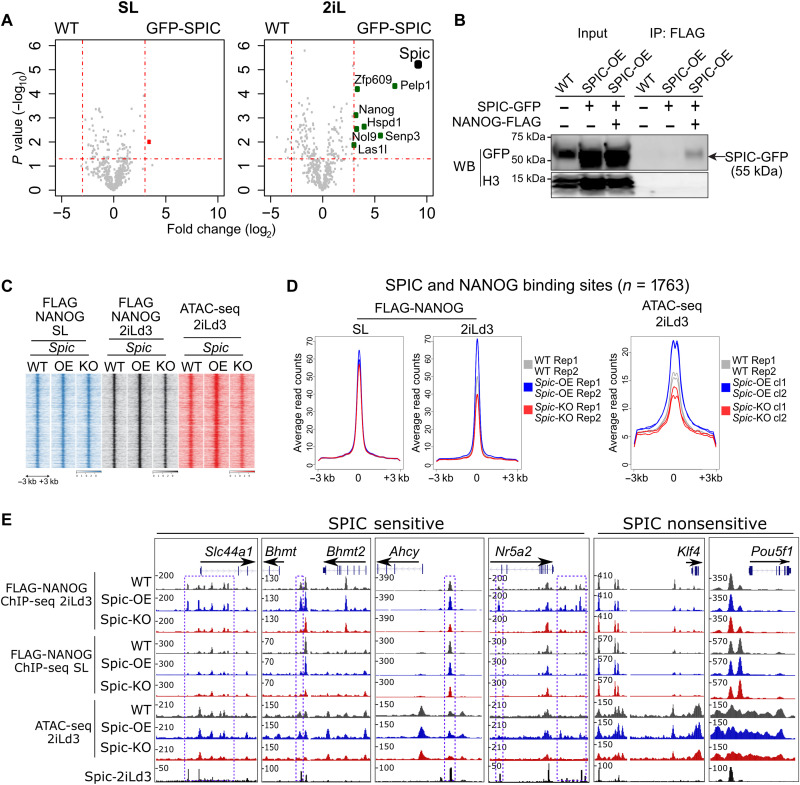
SPIC interacts with NANOG and stabilizes its chromatin binding. (**A**) IP-MS of GFP-SPIC showing the associated proteins in SL- and 2iLd3-ESCs. WT ESCs were used as negative control. Volcano plots show the log_2_ fold change and *P* value (based on false discovery rate–corrected *t* test) of iBAQ values in GFP-SPIC versus WT IPs based on *n* = 3 biological samples. (**B**) Immunoblot showing NANOG-SPIC interaction in 2iLd3-ESCs. ESCs stably expressing SPIC-GFP and FLAG-NANOG were used in FLAG-NANOG IP followed by immunoblotting for SPIC-GFP. WT ESCs and SPIC-GFP without FLAG-NANOG were used as controls. (**C**) Heatmap showing the chromatin binding of FLAG-NANOG and ATAC-seq signals in *Spic*-KO, *Spic*-OE, and *Spic*-WT ESCs cultured in SL or 2iLd3. For FLAG-NANOG, average ChIP-seq read counts of *n* = 2 biological replicates are shown. For ATAC-seq, average read counts of *n* = 2 independent clones of *Spic*-KO or *Spic*-OE and *n* = 2 biological replicates of WT ESCs are shown. (**D**) Average plot quantification of mean signals as shown in (C). (**E**) Genome browser view showing that SPIC influences NANOG binding at specific sites in 2iL-ESCs.

To better understand the role of SPIC in 2iL-ESCs, we mapped its protein interactions by label-free GFP immunoprecipitation followed by liquid chromatography–tandem mass spectrometry (LC-MS/MS) (IP-MS) in SPIC-GFP ESCs cultured in SL or 2iLd3 and used WT ESCs as negative controls (data S2). We detected several SPIC interactors in 2iL-ESCs, including the pluripotency TFs NANOG and ZFP609 (Zinc finger protein 609) and several members of the Five Friends of Methylated Chtop (5FMC) protein complex (*20*), including PELP1 (Proline, glutamate and leucine rich protein 1), SENP3 (SUMO specific peptidase 3), NOL9 (Nucleolar protein 9), and LAS1L1 (LAS1 like ribosome biogenesis factor 1) (Fig. 3A). The specificity of our IP-MS experiments is demonstrated by the absence of significant SPIC interactors in SL-ESCs, which do not express SPIC data (Fig. 3A). We further confirmed the interactions of top candidates by reverse IP using constructs expressing FLAG-NANOG or FLAG-PELP1 followed by immunoblotting for SPIC-GFP (Fig. 3B and fig. S2B).

As we detected a high co-occupancy of NANOG at SPIC binding sites (fig. S1D), we next assessed how SPIC affects NANOG binding to chromatin. We expressed FLAG-NANOG in *Spic*-WT, *Spic*-OE, and *Spic*-KO ESCs and examined NANOG chromatin binding by FLAG ChIP-seq (Fig. 3, C and D, and fig. S2C). Confirming our previous co-occupancy analysis, 74% of all SPIC binding sites overlapped at least one NANOG peak (*n* = 1763/2379 peaks; data S3). SPIC depletion or overexpression significantly affected NANOG binding in 44% of all SPIC-NANOG–bound regions [*n* = 788 peaks, *P* < 0.05, fold change (FC) > 1.5]. NANOG binding sites that are sensitive to SPIC depletion were mainly located near genes involved in choline/1C metabolism (e.g., *Slc44a1, Bhmt*, and *Ahcy*; fig. S3, A and B). At these sites, NANOG binding was not affected in SL-ESCs, indicating a SPIC-specific effect on 2iL-ESCs (Fig. 3E and fig. S3C). In line with differential NANOG binding, measuring chromatin accessibility by assay for transposase-accessible chromatin with sequencing (ATAC-seq) in SPIC-KO, SPIC-OE, and SPIC-WT ESCs demonstrated that *Spic* overexpression or depletion leads to increased and decreased chromatin accessibility at SPIC-NANOG binding sites, respectively (Fig. 3, D and E). Thus, SPIC stabilizes NANOG binding at specific regulatory elements that are mainly associated with choline/1C metabolism.

### SPIC regulates 1C metabolism

**Fig. 4.**
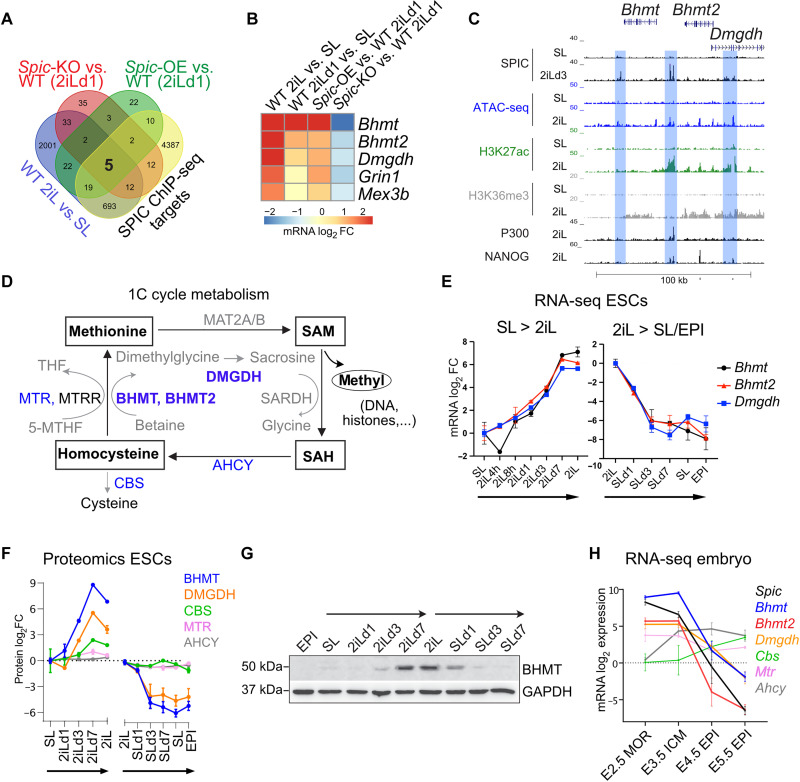
SPIC regulates 1C metabolism genes. (**A**) Venn diagram showing that five genes are differentially expressed in different comparisons and have SPIC binding in close vicinity. (**B**) Heatmap showing the mRNA log_2_ fold change (based on RNA-seq) of the five genes identified in (A). (**C**) Genome browser view showing SPIC binding and epigenetic marking of *Bhmt*, *Bhmt2*, and *Dmgdh* loci in SL-ESCs and 2iL-ESCs. (**D**) Schematic representation of the 1C metabolic cycle. (**E**) Graphs showing mRNA expression (based on RNA-seq) of *Bhmt*, *Bhmt2*, and *Dmgdh* during 2iL-SL-EPI transitions. The bars represent the means, and the error bars represent the SEM of *n* = 2 biological replicates. (**F**) Graphs showing protein expression [based on whole-cell proteomic (*15*)] of the 1C cycle genes during 2iL-SL-EPI transitions. Only proteins with detectable values in whole-cell proteomic are shown. The bars represent the means, and the error bars represent the SEM of *n* = 3 biological replicates. (**G**) Immunoblot showing BHMT protein expression during 2iL-SL-EPI transitions. (**H**) Graphs showing mRNA expression (based on RNA-seq) of the 1C cycle genes during early mouse embryonic development. The bars represent the means, and the error bars represent the SEM of *n* = 2 biological replicates.

To understand the role of *Spic* at the molecular level and to capture the early response to *Spic* perturbation, we cultured *Spic*-KO and *Spic*-OE ESCs in 2iL medium for 1 day (SL-to-2iLd1) and analyzed the transcriptional profile by RNA-seq and identified differentially expressed genes when compared to WT ESCs [false discovery rate (FDR) < 0.05 and FC > 2; fig. S3D and data S4]. As *Spic* is highly induced in 2iL-ESCs, we also analyzed the expression profiles of WT ESCs fully adapted in 2iL (cultured for over 21 days in 2iL) and selected genes that show concordant differential expression when compared to SL-ESCs (FDR < 0.05 and FC > 2). Last, to identify direct targets of SPIC, we selected genes that showed SPIC binding within 100 kb of vicinity of their promoters (Fig. 4A). This integrative analysis allowed us to focus on five high-confidence genes—*Bhmt* (*betaine-gomocystein S-methyltransferase*), *Bhmt2* (*betaine-homocystein S-methyltransferase 2*), *Dmgdh* (*dimethylglycine dehydrogenase*), *Grin1* (*glutamate ionotropic receptor NMDA type subunit 1*), and *Mex3b* (*Mex-3 RNA binding family member B*)—which represent the early and direct response to *Spic* in 2iL-ESCs (Fig. 4B).

**Fig. 5.**
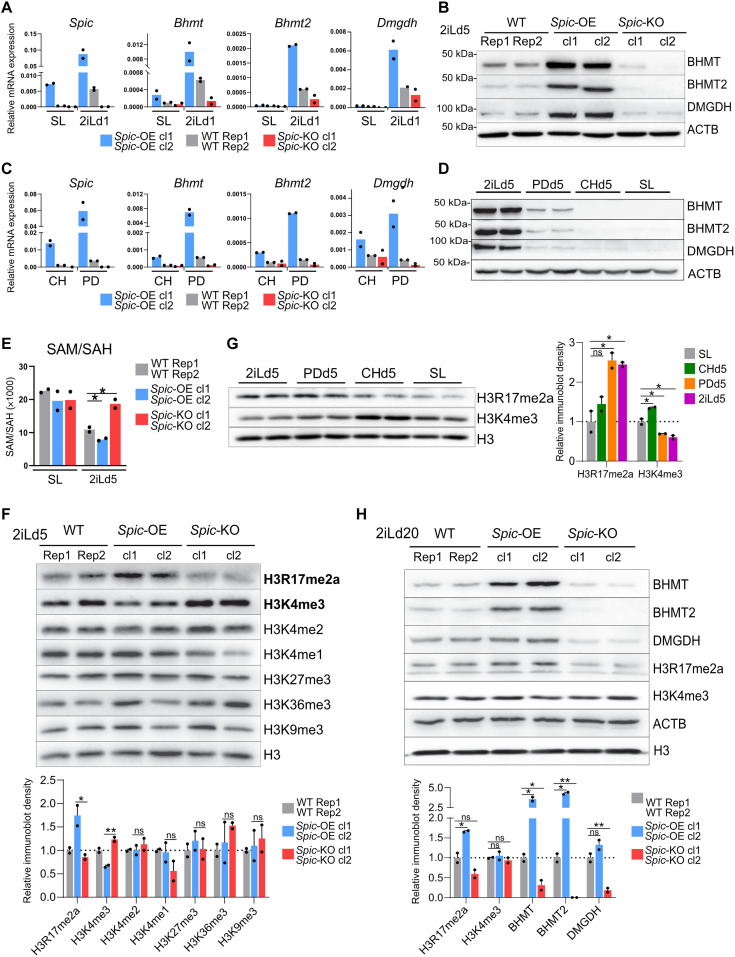
SPIC regulates SAM/SAH level and histone methylation. (**A**) qRT-PCR analysis showing expression of *Bhmt*, *Bhmt2*, and *Dmgdh* in *Spic*-KO, *Spic*-OE, and *Spic*-WT ESCs cultured in SL and 2iLd1.The bars represent the means of *n* = 2 independent clones of *Spic*-KO or *Spic*-OE and *n* = 2 biological replicates of WT ESCs. (**B**) Immunoblot showing expression of BHMT, BHMT2, and DMGDH in *Spic*-KO, *Spic*-OE, and *Spic*-WT 2iLd5-ESCs. Two independent clones of *Spic*-KO or *Spic*-OE and *n* = 2 biological replicates of WT ESCs are used. (**C**) qRT-PCR analysis showing expression of *Bhmt*, *Bhmt2*, and *Dmgdh* in *Spic*-KO, *Spic*-OE, and *Spic*-WT ESCs cultured for 5 days in PD0325901/LIF or CHIR99021/LIF medium. The bars represent the means of *n* = 2 independent clones of *Spic*-KO or *Spic*-OE and *n* = 2 biological replicates of WT ESCs. (**D**) Immunoblot showing expression of BHMT, BHMT2, and DMGDH in WT ESCs cultured for 5 days in PD0325901/LIF, CHIR99021/LIF, or 2iL-supplemented media. Two independent clones of *Spic*-KO or *Spic*-OE, and *n* = 2 biological replicates were used. (**E**) Graph showing the ratio of SAM/SAH as measured by MS in SL-ESCs and 2iLd5-ESCs. The bars represent the means of *n* = 2 independent clones of *Spic*-KO or *Spic*-OE and *n* = 2 biological replicates of WT ESCs. (**F**) Immunoblot and densitometry analysis showing levels of different methylated histone marks in *Spic*-KO, *Spic*-OE, and *Spic*-WT 2iLd5-ESCs. Two independent clones of *Spic*-KO or *Spic*-OE and *n* = 2 biological replicates of WT ESCs are used. (**G**) Immunoblot showing levels of H3K4me3 and H3R17me2a WT ESCs cultured in PD0325901/LIF or CHIR99021/LIF medium. *n* = 2 biological replicates. (**H**) Immunoblot and densitometry analysis showing the levels of H3R17me2a, H3K4me3, BHMT, BHMT2, and DMGDH in long-term 2iL-cultured *Spic*-KO, *Spic*-OE, and *Spic*-WT ESCs. Two independent clones of *Spic*-KO or *Spic*-OE and *n* = 2 biological replicates of WT ESCs are used. In all graphs, the error bars show the SEM and the asterisks represent *P* < 0.05, unpaired two-tailed Student’s *t* test.

The top three *Spic* targets—*Bhmt*, *Bhmt2*, and *Dmgdh*—are clustered in close proximity in the genome and share multiple SPIC binding sites at adjacent enhancers (Fig. 4C). Furthermore, *Bhmt*, *Bhmt2*, and *Dmgdh* have related functions in 1C metabolism (Fig. 4D). The 1C metabolic cycle is used in most cells for conversion of homocysteine to methionine by methionine synthase (MTR) and using methyl groups carried by derivates of folic acid. However, in liver and early embryo, the conversion of homocysteine to methionine is also supported by sequential activity of BHMT/BHMT2 and DMGDH that use betaine as methyl group donor (*21*). Similar to *Spic*, we observed that *Bhmt*, *Bhmt2*, and *Dmgdh* are highly induced in 2iL-ESCs and are rapidly down-regulated during 2iL- to SL-EPI transition (Fig. 4, E to G). In addition, *Bhmt*, *Bhmt2*, and *Dmgdh* are highly expressed in preimplantation embryo and are down-regulated in the postimplantation epiblast (Fig. 4H) (*22*). To establish a direct link with *Spic*, we measured the level of *Bhmt*, *Bhmt2*, and *Dmgdh* in *Spic-*KO *and Spic-*OE ESCs and found that depleting *Spic* suppresses *Bhmt*, *Bhmt2*, and *Dmgdh* expression, whereas *Spic*-OE elevates the level of these genes in 2iL-ESCs (Fig. 5, A and B). As *Spic* is induced downstream of PD0325901 treatment (inhibition of MEK/ERK signaling), we also measured the levels of *Bhmt*, *Bhmt2*, and *Dmgdh* in cells treated with PD0325901 or CHIR99021 and found that these genes are highly induced downstream of MEK/ERK inhibition (Fig. 5, C and D). Notably, betaine is not supplemented in ESC cultures but can be produced from choline, which is present in both 2iL and SL media. Consistent with our findings, the choline transporter, *Slc44a1*, is up-regulated in 2iL-ESCs, indicating that 2iL-ESCs have a higher capacity for choline uptake (fig. S3E). Together, these findings demonstrate that *Spic* directly activates the 1C metabolism genes *Bhmt*, *Bhmt2*, and *Dmgdh* in 2iL-ESCs.

The 1C metabolic cycle plays a central role in epigenetic regulation by controlling the levels of SAM and *S*-adenosyl-l-homocysteine (SAH). Therefore, we asked whether *Spic* affects the SAM/SAH production in ESCs. Using MS, we measured cellular levels of SAM and SAH and found that *Spic* overexpression results in increased SAM-to-SAH flux, whereas *Spic* depletion leads to decreased SAM-to-SAH conversion in 2iL-ESCs (Fig. 5E). SAM is a universal donor of the methyl group for DNA, histone, RNA, lipid, and protein methylation. Therefore, we asked whether *Spic* influences the level of DNA methylation. Using MS, we measured the levels of 5-methylcytosine (5mC) and 5-hydroxymethyl cytosine (5hmC) in *Spic*-OE and *Spic*-KO ESCs and found that *Spic* has no effect on 5mC and 5hmC levels (fig. S4A). We next asked whether *Spic* affects histone methylation and analyzed a panel of methyl histone marks by Western blot. We found that *Spic* specifically reduces the level of H3K4me3 and increases the level of H3R17me2 in 2iL-ESCs but not SL-ESCs (Fig. 5F and fig. S4B). Accordingly, treatment with PD0325901 (MEK/ERK inhibition) reduced the level of H3K4me3 and increased the level of H3R17me2, further linking *Spic* and the 1C metabolism genes to these specific epigenetic changes (Fig. 5G). These findings were further confirmed by additional antibodies against H3K4me3 and H3R17me2 marks (fig. S4, C and D).

We validated our findings in long-term 2iL-cultured ESCs (>day 20) and confirmed that SPIC is essential for the expression of 1C metabolism genes. This was assessed through quantitative reverse transcription polymerase chain reaction (qRT-PCR) and immunoblotting for *Bhmt*, *Bhmt2*, and *Dmgdh* (Fig. 5H and fig. S4E), as well as the examination of SPIC chromatin binding at these loci in long-term 2iL-ESCs (fig. S4F). Additional RNA-seq analysis demonstrated higher levels of a set of naïve markers in *Spic*-OE compared to *Spic*-KO (fig. S5, A and B). Consistent with our previous results, *Spic*-OE ESCs exhibited reduced SAM/SAH and elevated levels of H3R17me2a compared to *Spic*-KO ESCs in steady-state 2iL-ESCs (Fig. 5H and fig. S5C). However, no significant differences were observed in H3K4me3 levels among the different *Spic*-ESCs, suggesting that the level of H3K4me3 can be regulated by additional pathways during 2iL adaptation (Fig. 5H).

**Fig. 6.**
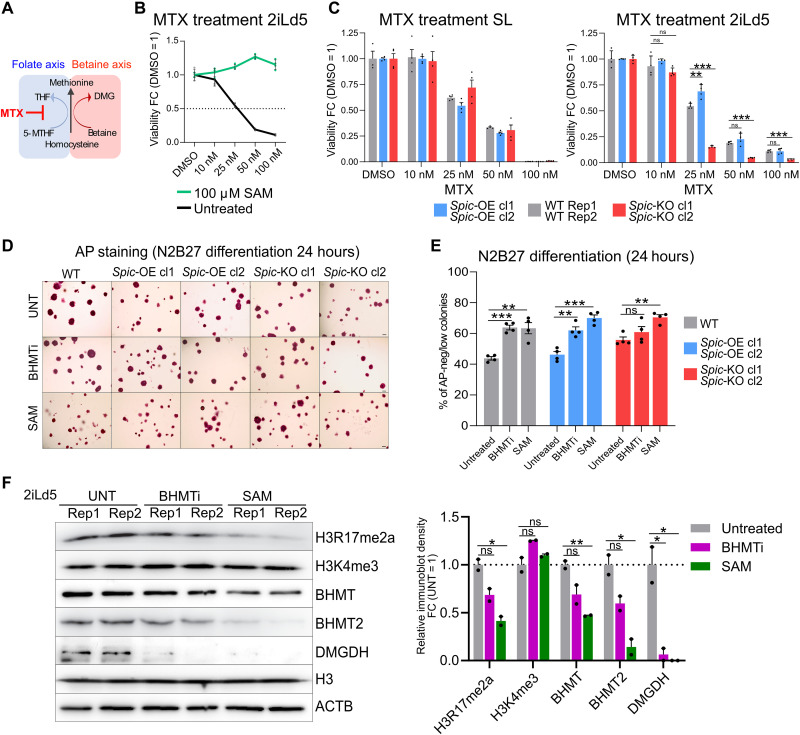
Targeting 1C metabolism affects ESCs differentiation. (**A**) Schematic representation of the two pathways involved in driving 1C metabolism. MTX specifically blocks the folate pathway. (**B**) MTX response in WT ESCs cultured for 5 days in 2iL with or without SAM supplementation. The lines represent the mean of DMSO (dimethyl sulfoxide)–normalized viability of *n* = 2 biological replicates. (**C**) MTX response in 2iLd5 *Spic*-KO, *Spic*-OE, and *Spic*-WT ESCs. The bars represent the mean viability of two independent clones and *n* = 2 biological replicates per clone for *Spic*-KO and *Spic*-OE ESCs and *n* = 4 biological replicates for WT ESCs. (**D** and **E**) AP staining in *Spic*-KO, *Spic*-OE, and *Spic-*WT ESCs treated with SAM or BHMTi (CBHcy) for 5 days during SL-to-2iLd5 transition, followed by 24 hours of differentiation in N2B27 medium. Scale bars, 200 μm. Barplot represents the percentage of AP-low colonies upon N2B27 differentiation under different in each treatment condition. Two independent clones and *n* = 2 biological replicates per clone were used for *Spic*-KO and *Spic*-OE ESCs and *n* = 3 biological replicates for WT ESCs. (**F**) Immunoblot and the corresponding densitometry analysis showing the levels of BHMT, BHMT2, DMGDH, H3R17me2a, and H3K4me in WT ESCs treated with SAM or BHMTi (CBHcy) for 5 days during SL-to-2iLd5 transition. The bars show the mean of *n* = 2 biological replicates per treatment condition. In all graphs, the error bars show the SEM and the asterisks represent *P* < 0.05, unpaired two-tailed Student’s *t* test.

**Fig. 7.**
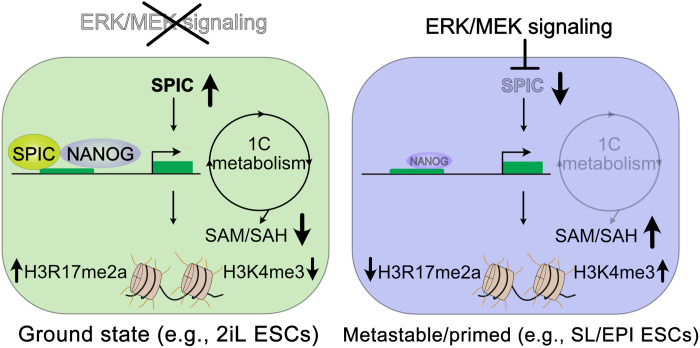
*Spic* links cellular metabolism to epigenetic regulation in ground-state pluripotency. *Spic* is induced in response to MEK/ERK inhibition in 2iL-ESCs and is up-regulated in early blastocyst. SPIC stabilizes NANOG binding and increases chromatin accessibility at genes involved in choline and 1C metabolism. By activating the betaine-dependent 1C metabolism, SPIC increases SAM-to-SAH flux, maintains low levels of SAM, and controls the level of H3K4me3 and H3R17me2 histone methylation in ground-state ESCs. During transition from ground-state to primed-state pluripotency, ERK/MEK signaling represses *Spic* and 1C metabolism.

The cooperation between the betaine- and folate-dependent pathways regulates 1C metabolism (Fig. 6A). Therefore, we asked whether SPIC influences the reliance of ESCs on the folate pathway. We used methotrexate (MTX), a known inhibitor of the folate pathway, and assessed the responsiveness of *Spic*-KO, *Spic*-OE, and WT ESCs to MTX treatment. Notably, the detrimental effects of MTX can be fully rescued by supplementing ESCs with SAM (Fig. 6B). We found that depleting *Spic* enhances the susceptibility of ESCs to MTX treatment (Fig. 6C). Similarly, SL-ESCs, which exhibit lower betaine-dependent 1C metabolism, demonstrated higher sensitivity to MTX when compared to 2iL-ESCs (fig. S5D). These findings suggest that activation of the betaine-dependent 1C metabolism downstream of *Spic* enhances the robustness of pluripotent cells against environmental perturbations affecting the folate pathway. Last, we manipulated the intracellular SAM/SAH levels using treatment with either the BHMT inhibitor (BHMTi; CBHcy) (*23*) or by supplementing ESCs with exogenous SAM. Both treatments phenocopy *Spic*-KO ESCs: CBHcy inhibits BHMT activity, leading to a reduced SAM-to-SAH flux, while SAM supplementation directly increased the SAM/SAH ratio. We observed that treatment with BHMTi or SAM leads to accelerated ESC differentiation as measured by reduced number of AP-positive colonies and decreased expression of naïve pluripotency markers (Fig. 6, D and E, and fig. S5E). Both BHMTi and SAM treatments also reduced the level of H3R17me2a, although this effect was more pronounced on SAM treatment (Fig. 6F). Together, our findings demonstrate that *Spic* regulates 1C metabolism, leading to decreased SAM/SAH ratio and increased level of H3R17me2a histone mark in ground-state pluripotency (Fig. 7).

## DISCUSSION

In this study, we report *Spic* as a specific marker of ground-state pluripotency. We found that *Spic* is not essential for ESC maintenance but can attenuate the exit from naïve pluripotency. These observations are in line with the phenotype of *Spic*-null mice that are viable and fertile but show increased embryonic lethality and are born at lower-than-expected Mendelian ratios (9% versus 25%) (*9*). Furthermore, RNA interference of *Spic* in early mouse embryo impaired early development, resulting in only ~60% of embryos developing to the blastocyst stage (*24*).

We found that SPIC acts as an auxiliary TF to stabilize NANOG binding at loci associated with choline and 1C metabolism. Notably, we observed basal NANOG binding and chromatin accessibility at *Bhmt*, *Bhmt2,* and *Dmgdh* loci in SL-ESCs, suggesting that recruitment of SPIC is essential for full activation of the loci in 2iL-ESCs. We also observed interactions between SPIC and members of the 5FMC complex, which is involved in protein deSUMOylation (*20*), suggesting that recruitment of 5FMC may influence TF SUMOylation and binding at SPIC-NANOG binding sites.

We found that *Spic* activates 1C metabolism in 2iL-ESCs. Consistent with our findings, levels of betaine, SAH, folate, and methionine are elevated at morula stage, suggesting that the 1C cycle is activated during morula to blastocyst transition (*25*, *26*). Similar to ESCs, the betaine transporters, SIT1 (Slc6a20a) and PROT (Slc6a7), are not expressed in blastocysts, and betaine is mainly produced via choline catabolism (*27*). In addition, BHMT is highly active in blastocysts, which coincides with choline consumption and SAH accumulation (*25*, *26*). In line with these observations, knockdown of *Bhmt* in early embryos impairs blastocyst development and reduces the number of cells in ICM, a defect that can be rescued by exogenous methionine (*26*).

We show that *Spic* maintains low levels of SAM and decreases the SAM/SAH ratio in 2iL-ESCs. Consistently, reduced level of SAM has been observed in naïve ESCs and in blastocyst-stage embryos (*28*, *29*). Furthermore, treatment with homocysteine can reduce SAM levels and the methylation index in ESCs (*28*). SAM serves as a universal methyl group donor, participating in the methylation of various target macromolecules. The high consumption rate of SAM in naïve ESCs may account for the observed elevated SAM-to-SAH flux (*30*). In line with this, previous studies demonstrated that choline can stimulate methionine production without causing a significant accumulation of SAM. This is due to the rapid utilization of SAM in other cellular processes such as carnitine production and fatty acid oxidation (*31*). Cells with impaired phosphatidylethanolamine methylation exhibit an accumulation of SAM (*32*). Here, we observed one potential transmethylation substrate, H3R17me2a, which is increased in 2iL-ESCs downstream of *Spic*. H3R17me2a is a key histone modification associated with active transcription (*33*, *34*) and determines the ICM fate in early embryos (*35*). Accordingly, depletion of CARM1 (Coactivator associated arginine methyltransferase 1) and PRMT6 (Protein arginine methyltransferase 6), which deposit H3R17me2a, hinders embryonic development (*36*) and leads to mESCs differentiation (*37*, *38*).

We found that decreased SAM/SAH ratios downstream of *Spic* are associated with decreased levels of H3K4me3. This is in line with previous studies showing a direct relationship between SAM and H3K4me3 levels (*39*, *40*) and that the global levels of H3K4me3 increase in SL-ESCs versus 2iL-ESCs (*41*) and in the embryonic day 6.5 (E6.5) postimplantation versus preimplantation epiblast (*42*), where *Spic* is strongly repressed.

Plausible direct or indirect mechanisms can explain the opposite response of H3K4me3 and H3R17me2a to SPIC. The H3K4 methyl transferases (MLL/SET) have a higher Michaelis constant (*K*_m_) for SAM (i.e., optimum enzymatic activity at a higher SAM concentration) compared to some other methyltransferases such as PRMT4 (CARM1) (*43*, *44*). For example, when histone H3 is used as a substrate, PRMT4 has a *K*_m_ of 0.21 ± 0.052 for SAM, while MLL2, a key methyltransferase for H3K4me3 deposition in mESCs, exhibits a *K*_m_ of 3.17 ± 0.37 (*44*). Consequently, the reduced SAM levels in response to *Spic* may preferentially decrease H3K4me3. In addition, SAH directly inhibits H3K4 methyltransferases activity (*29*, *39*, *45*), offering another potential mechanism through which SPIC specifically decreases H3K4me3 levels. Last, H3R17me2a is linked to naïve pluripotency, whereas increased H3K4me3 is associated with ESC differentiation (*41*, *42*). Thus, we cannot dismiss the possibility that the increased H3R17me2a and decreased H3K4me3 levels indirectly reflect a global shift of ESCs toward a more naïve state of pluripotency. Further investigation is required to gain better understanding of how SPIC regulates histone methylation in pluripotent cells.

*Spic* plays an instructive role in macrophage and B cell differentiation. Mechanistically, *Spic* is derepressed by heme, leading to differentiation of splenic RPM and bone marrow macrophages (*8*, *9*). *Spic* is also induced in response to inflammatory signals in monocytes and macrophages (*13*), leading to increased iron efflux and restrain of the inflammatory response (*13*, *14*). In this study, we show additional roles of *Spic* in mESCs; whether regulation of betaine-dependent 1C metabolism downstream of *Spic* plays a role in immune cells under specific conditions such as inflammation remains to be investigated. Together, our results expand the compendium of TFs involved in ground-state pluripotency and link cellular metabolism to epigenetic regulation in ESCs via the function of *Spic*.

## MATERIALS AND METHODS

### Cell culture

mESCs (E14Tg2a) were routinely cultured under SL condition, on 0.1% gelatin-coated plates. To make SL medium, high-glucose Dulbecco’s modified Eagle’s medium (Gibco) was supplemented with 14% heat-inactivated fetal bovine serum (Gibco), 1% GlutaMAX (Gibco), 1% Na pyruvate (Gibco), 1% penicillin/streptomycin (Gibco), 55 μM β-mercaptoetanol, and LIF (1000 U ml^−1^’; Millipore). Under serum-free 2iL condition, cells were cultured on gelatin-coated dishes in NDiff 227 (TaKaRa) medium supplemented with penicillin/streptomycin, LIF, and two inhibitors, GSK3 inhibitor CHIR99021 (3 μM; Axon) and MEK inhibitor PD0325901 (1 μM; Axon). For single-inhibitor treatment experiments, only the noted inhibitor was omitted from the complete 2iL medium. To induce EpiLSC differentiation, 2iL cells were seeded on fibronectin (10 μg ml^−1^; Millipore)–coated plates at a density of 15,000 cells/cm^2^ in NDiff 227 medium supplemented with 1% knockout serum replacement (Gibco), Activin-A (20 ng/ml; PeproTech), basic FGF (12 ng ml^−1^; PeproTech), and 1% penicillin/streptomycin. Exit from pluripotency was induced by culturing the 2iL-adapted cells in NDiff 227 medium without PD0325901, CHIR99021, and LIF for 24 hours. Treatment of ESCs with SAM and BHMTi was performed for 5 days and using 100 μM SAM (Sigma-Aldrich) or 50 μM BHMTi CBHcy (AOBIOUS) before ESCs were used in further experiments.

### Generating *Spic-*KO and *Spic*-OE ESCs

CRISPR-Cas9–mediated genome editing was used to knock out *Spic* in mESC. Two guide RNAs (gRNAs) were designed to target Spic exon 4 and 5 and were cloned into pSpCas9(BB)-2A-Puro (Addgene) using Bbs I digestion. E14 cells were transfected with the recombinant Cas9 and gRNA plasmids using Lipofectamine 3000 (Invitrogen) according to the manufacturer’s instruction. Transfected cells were selected through treating with puromycin (1 μg/ml; Gibco) for 24 hours and cultured in clonal density afterward. Individual colonies were expanded and subjected to DNA isolation. PCR was used to assess correct edits and homozygosity. *Spic*-OE clones were generated by transfecting E14 cells with BAC containing SPIC-GFP expression cassette under *Spic* endogenous regulatory elements. Positive clones where selected by geneticin (400 μg/ml; Gibco) treatment and further clonal expansion. *Spic* overexpression was confirmed by qRT-PCR and by GFP immunoblotting. Two independent clones for *Spic*-KO or *Spic*-OE ESCs were used in all experiments.

### Proliferation and viability assays and AP staining

Cell proliferation was assessed in SL and at different time points of transition to 2iL cultures. Cell proliferation was measured using CellTiter-Glo (Promega) over a period of three consecutive days for each specific time point. To determine the response of ESCs to MTX, cells were treated with different doses of MTX (Sigma-Aldrich) for 48 hours, and cell viability was examined using the CellTiter-Glo (Promega). AP activity was assessed using the Alkaline Phosphatase Detection Kit (Sigma-Aldrich). Briefly, mESCs were cultured at clonal density (500 cells per well) in 24-well plates for 5 days under the noted experimental conditions followed by induction of differentiation in N2B27 medium. Colonies were fixed with 4% paraformaldehyde and stained for AP activity following the manufacturer’s instructions. The number of AP-low colonies was calculated by subtracting the number of AP-positive colonies from the total number of colonies, and the data were presented as a percentage of the total colonies.

### Genomic DNA isolation

For PCR validation of CRISPR-Cas9 experiments, cells were harvested, washed with phosphate-buffered saline (PBS), and lysed in 500 μl of lysis buffer [10 mM tris (pH 7.5), 10 mM EDTA, 10 mM NaCl, and 0.5% sarcosyl]. Proteinase K (100 μg/ml; Invitrogen) was added to the lysate and samples were incubated at 55°C overnight. The next day, the same volume of ice-cold isopropanol was mixed with the lysate, and DNA was precipitated by centrifuging the samples at 12,000*g* for 15 min. DNA pellet was washed with 70% ethanol and resuspended in nuclease-free water. For DNA methylation analysis, DNA isolation was performed using the Wizard Genomic DNA Purification kit (Promega) according to manufacturer’s instructions.

### DNA (hydroxy)methylation analysis of nucleotides

For DNA methylation analysis, 150 to 1000 ng of genomic DNA was digested using DNA Degradase Plus (Zymo Research). Digested nucleosides were then analyzed using a high-performance LC-MS/MS system consisting of an Acquity UPLC (Waters, Milford, MA), a Waters Atlantis Hilic column (2.1 mm by 100 mm, 3 μm), and Micromass Quattro Premier XE (Waters).

### LC-MS for SAM/SAH detection

Samples were processed as described before (*46*–*48*). Briefly, about 10 million ESCs were dissociated with trypsin, and cell pellet was washed twice with PBS and dissolved in 500 μl of water, vortexed, and deproteinized by the addition of perchloric acid. Samples were centrifuged for 10 min at 2000*g*, and the supernatant was collected. For long-term 2iL samples, cell pellet was homogenized in 0.2 ml of ice-cold 0.091 mM acetic acid. An internal standard mixture containing 0.50 μM ^13^C_5_-SAH and 1.0 μM ^2^H_3_-SAM was added to each sample to normalize for variations in extraction performances. The solution was neutralized by 1 M phosphate buffer (pH 11.5) and was applied to an OASIS solid-phase extraction column (60 mg, 3 ml; Waters) preconditioned with 1 ml of methanol, 750 μl of 10 mM lauric acid in 0.1 M NaOH, and 1 ml of H_2_O. Samples were then eluted from the column with 800 μl of H_2_O-methanol (85:15, v/v), containing formic acid (1 ml/liter). LC-MS/MS was performed on a Sciex 6500 triple quadrupole tandem mass spectrometer (AB Sciex), consisting of an Exion UPLC pump and an Exion autosampler. Data were acquired and processed using Analyst for Windows NT software (version 1.7.1).

### Generating FLAG-NANOG and FLAG-PELP1 expressing ESCs

*Pelp1* open reading frame was amplified from mESC cDNA using Phusion polymerase (Thermo Fisher Scientific), fused to 3XFlag coding sequence by overlapping PCR and further cloned into pPy-CAG-Cre-ERT2-IRES-BSD (Addgene). The recipient plasmid and the 3Xflag-Pelp1 PCR amplicon were digested with Age I [New England Biolabs (NEB)] and Not I (NEB), ligated using T4 DNA ligase (Invitrogen), and finally transformed into DH10B competent cells (Invitrogen). Colonies containing recombinant plasmids were selected using ampicillin (100 μg/ml) LB-agar culture. FLAG-NANOG–expressing plasmid was obtained from I. Chambers, University of Edinburgh (*49*). *Spic*-KO and *Spic*-OE clones and E14 cells were further transfected with FLAG-NANOG– and FLAG-PELP1–expressing plasmids. Transfected ESCs were treated with puromycin (1 μg/ml for 4 days; Gibco) or blasticidin (10 μg/ml for 3 days; InvivoGen) to select for FLAG-NANOG and FLAG-PELP1, respectively. Expression of tagged proteins was further confirmed by immunoblotting.

### RNA isolation and qRT-PCR

RNA extraction was done using an RNeasy mini kit (QIAGEN) and according to the manufacturer’s instructions. Isolated RNA was quantified with Nanodrop (Thermo Fisher Scientific), and 1 μg of RNA was used in cDNA synthesis reaction using Superscript III (Invitrogen) and a mix of oligo dT and random hexamers according to manufacturer’s instructions. cDNA was further diluted 1:10 and was used for qRT-PCR using the iTaq Universal SYBR Green Supermix (Bio-Rad).

### RNA-seq and data analysis

RNA-seq libraries were prepared using 1 μg of total RNA isolated with the RNeasy kit (QIAGEN) and using the KAPA RNA HyPerPrep Kit with RiboErase for rRNA depletion (Roche) according to the manufacturer’s instructions. RNA-seq in long-term 2iL-ESCs was performed using a Novogene mRNA-seq service. The final libraries were sequenced on a HiSeq-2000 Illumina sequencer. To analyze the RNA-seq data, FASTQ reads were mapped against mouse mm9 reference genome using STAR (*50*) and default setting. Mapped reads were assigned to RNAs using HTseq-count (*51*) with the following settings: -m union -s reverse (for RiboErase RNA-seq) -t exon. Samples were then normalized for library size using DESeq2 (bioconductor.org) (*52*), and values were further used for differential gene expression analysis with the following criteria: minimum read number > 50 reads per gene in at least one sample, FDR < 0.05, and FC > 1.5. Graphs were generated in R version 3.5.1 on Ubuntu 16.04.5 LTS.

### Chromatin immunoprecipitation

mESCs were cultured in SL or 2iL medium for 3 or 25 days. For each replicate, 15 million cells were fixed with 1% paraformaldehyde. Fixed cells were consecutively treated with buffer A (148 mM NaCl, 1.48 mM EDTA, 0.74 mM EGTA, and 74 mM Hepes), buffer B (10 mM EDTA, 0.5 mM EGTA, 20 mM Hepes, and 0.25% Triton X-100), and buffer C (150 mM NaCl, 1 mM EDTA, 0.5 mM EGTA, and 50 mM Hepes). Isolated nuclei were lysed in freshly made sonication buffer (150 mM NaCl, 1 mM EDTA, 0.5 mM EGTA, 20 mM Hepes, 0.15% SDS, 1% Triton X-100, and 1× protease inhibitor), and samples were sonicated using Bioraptor Pico (Diagenode). Sonicated chromatin was precleared using bovine serum albumin (BSA)–blocked protein A/G beads (Invitrogen) and was further incubated with 10 μg of primary antibody (anti-FLAG from Sigma-Aldrich for NANOG ChIP and anti-GFP from Abcam for SPIC-GFP ChIP). After overnight incubation, the immune complexes were captured by incubation with BSA-blocked protein A/G beads for 2 hours on a rotating wheel at 4°C. Beads were washed with ChIP wash buffer 1 [2 mM EDTA, 20 mM tris (pH 8), 150 mM NaCl, 1% Triton X-100, and 0.1% SDS], buffer 2 [2 mM EDTA, 20 mM tris (pH 8), 150 mM NaCl, 1% Triton X-100, 0.1% SDS, and 500 mM NaCl], and buffer 3 [1 mM EDTA and 10 mM tris (pH 8)] for 10 min. Samples were then incubated with fresh ChIP elution buffer (0.1 M NaHCO_3_ and 1% SDS) for 30 min at room temperature. Eluted chromatin was de–cross-linked overnight by adding 5 M NaCl and proteinase K (final 10 mg/ml) and by incubating at 65°C. DNA was purified using the MinElute PCR Purification Kit (QIAGEN) and was subjected to library preparation using the KAPA HyperPrep kit (Roche) according to the manufacturer’s instructions with dual indices and sequencing on an Illumina HiSeq 2500 sequencer [dual-indexed 50–base pair (bp) paired-end reads].

### Assay for transposase-accessible chromatin with sequencing

For ATAC-seq experiment 50,000 mESCs were used. Cells were cultured in 2iL medium for 3 days and were >90% viable before harvesting. Cells were dissociated with trypsin (Gibco) and were washed with PBS and equilibrated in the ATAC resuspension buffer [10 mM tris-HCl (pH 7.5), 10 mM NaCl, and 3 mM MgCl_2_]. Cells were incubated in 100 μl of lysis buffer [ATAC resuspension buffer plus 0.1% NP-40, 0.1% Tween 20, and 0.01% digitonin (Promega)] for 3 min on ice. The lysis reaction was diluted by adding 1 ml of resuspension buffer supplemented with 0.1% of Tween 20, and nuclei were pelleted by spinning at 900*g* for 7 min. Tagmentation reaction was set up by adding adaptor-loaded transposon 5 (Illumina) and Tagment DNA buffer (Illumina) to the permeabilized nuclei, and samples were incubated for 30 min at 37°C. DNA was purified using the MinElute PCR purification kit. NEBNext High-Fidelity 2X PCR Master Mix (NEB) and UDI primers for tagmented libraries (Diagenode) were used for library preparation. Libraries were preamplified for 4 cycles and subjected to qPCR to calculate the additional number of cycles needed to reach 25% of saturating amplification.

### ChIP-seq and ATAC-seq data analysis

FASTQ reads were mapped against mouse mm9 reference genome using Bowtie2 (*53*) Reads were filtered for high-quality mapping with SAMtools *q* > 15 (*54*), and PCR duplicates were removed using PicardTools. SAM files were converted to BAM using SAMTools (*54*). BAM files were converted to BED files using BEDTools (*55*), and reads were extended by 160 nt and converted to BedGraph using the bamCoverage tool from the deepTools suite (*56*). BedGraphs were converted to BigWig files using bedGraphToBigWig from the UCSC tool suite for further visualization using the Integrative Genomics Viewer browser. Peak calling was performed using MACS2 (*57*), and assigned peaks were filtered against the ENCODE black list regions. For read counting in assigned peaks, BAM files were first converted to BED files and read counting was performed using BEDTools (*55*). Read count across different samples were normalized for library size using DESeq2 (bioconductor.org) (*52*), and values were used for differential peak analysis with the following criteria: minimum read number > 20 reads per peak in at least one sample, *P* < 0.05, FC ≥ 1.5. To identify genes in the vicinity to ChIP-seq/ATAC-seq peaks, BEDTools window with -w 100,000 was used. For heatmap and average profile analysis, fluff (*58*) was used to compute values in 100 bp bins flanking the peak center by 5 kb. For motif analysis in SPIC ChIP-seq data, Homer (*59*) was used. Gene list enrichment analysis was done using EnrichR using default settings (*60*).

### Co-immunoprecipitation

E14^(*Spic*-OE, PELP1 or NANOG FLAG)^, E14^(*Spic*-KO PELP1 or NANOG FLAG)^, E14 ^(PELP1 or NANOG FLAG)^, and E14^(*Spic*-OE)^ ESCs were cultured in 2iL medium for 3 days. Approximately 30 million cells per IP reaction were collected and lysed in the TNE buffer (50 mM tris-HCl, 1 mM EDTA, 300 mM NaCl, 0.5% NP-40, and protease inhibitor). The lysate was incubated on ice for 15 min and then sonicated for 3 cycles (high power, 30 s on/30 s off). To remove the RNA/DNA-mediated interactions, 1 μl of benzonase (Merck) was added to the sonicated lysate. After a 15 min incubation on ice, the lysate was cleared by spinning at 17,000*g* for 30 min at 4°C. Fifty microliters of anti-Flag M2 Agarose beads (Sigma-Aldrich) was washed and blocked with 1% BSA for 1 hour at 4°C. Beads were washed with the TNE buffer and incubated with 16 mg of prepared protein lysate for 2 hours at 4°C. Beads were washed three times with the TNE buffer and two times with PBS. Beads were then boiled for 3 min in nonreducing sample buffer [50 mM tris (pH 6.8), 2% SDS, 10% glycerol, and 0.002% bromophenol blue] to release the captured proteins. To investigate the interaction between PELP1 or NANOG and SPIC, the eluted fraction was used in immunoblotting.

### Immunoblotting

Cells were lysed in radioimmunoprecipitation assay buffer (50 mM tris-HCl, 0.5 M pH 7.4, 150 mM NaCl, 1% NP-40, 1% Na-deoxycholate, 0.1% SDS, and 1 mM EDTA) supplemented with cOmplete Protease Inhibitor Cocktail (Sigma-Aldrich). Protein lysates were quantified with Pierce BCA Protein Assay Kit (Invitrogen) (and 50 μg was loaded on 8 or 12% SDS–polyacrylamide gel electrophoresis and transferred onto a polyvinylidene difluoride membrane. Blots were blocked with 5% nonfat milk and incubated with corresponding primary antibodies overnight at 4°C and then with suitable horseradish peroxidase–labeled secondary antibodies for 1 hour at room temperature. SuperSignal West Pico PLUS Chemiluminescent Substrate (Invitrogen) was used for signal detection. All antibodies (reagent list) were used in 1:1000 dilution except for anti-H3R17me2a, which was 1:500, and secondary antibodies that were diluted 1:2000.

### GFP pull-down followed by LC-MS/MS

To investigate SPIC interactions, ESCs expressing SPIC-GFP BAC were used under SL or 2iLd3 conditions and were compared to WT control ESCs. For each experimental group, three biological replicate pull-downs were used. Briefly, cells were dissociated using trypsin and washed with PBS. Cell pellet was resuspended in buffer A [10 mM Hepes-KOH (pH 8.0), 1.5 mM MgCl_2_, and 10 mM NaCl], incubated on ice for 15 min, and centrifuged for 5 min, 400*g*, at 4°C to remove the supernatant. Nuclei was separated using a Dounce homogenizer in hypotonic buffer (10 mM NaCl, 1.5 mM MgCl_2_, 10 mM Hepes-KOH, 0.15% NP-40, and protease inhibitor). Nuclei were pelleted by spinning at 3500*g* for 15 min, washed with PBS, and then lysed in lysis buffer [20 mM Hepes-KOH (pH 8.0), 2 mM MgCl_2_, 420 mM NaCl, 20% glycerol, 0.2 mM EDTA, 0.1% NP-40, 0.5 mM dithiothreitol (DTT), and protease inhibitor] for 1 hour at 4°C on a rotating wheel. Lysates were centrifuged for 45 min, 20,000*g* at 4°C, and the supernatant nuclear extract was collected. Two milligrams of the nuclear extract was used per pull-down and was diluted with lysis buffer without salt to reach the final concentration of 300 mM NaCl. Samples were incubated with ChromoTek GFP-Trap Agarose beads (Proteintech), and EtBr (0.05 mg/ml) was added to the samples to release the DNA-mediated interactions. Samples were incubated for 90 min on a rotating wheel at 4°C. Beads were washed twice with the lysis buffer containing 0.5% NP-40, then with PBS plus 0.5% NP-40, and finally twice with PBS. Beads were eluted using 2 M urea in 100 mM tris (pH 7.5) with 10 mM DTT for 20 min at room temperature. Samples were then digested with trypsin/LysC combination using Filter Aided Sample-Preparation as described previously (*61*).

### Analysis of MS/MS protein interaction data

Thermo Raw mass spectra were analyzed using MaxQuant 1.5.1.0 to search against the mouse RefSeq protein sequence and using default MaxQuant settings: 1% FDR, a minimum peptide length of seven amino acids was set, up to two missed cleavages sites, match-between-runs, label-free quantification, and iBAQ (intensity based absolute quantification) quantification of proteins enabled (*62*, *63*). We used the proteingroups.txt output table in Perseus (version 1.5.5.3) (*64*). We filtered out proteins that were flagged as “reverse” or “contaminant” from the final list using a decoy database from MaxQuant. Next, we filtered for proteins that have at least two detected peptides in which at least one is a unique peptide. We then selected proteins that were reproducibly quantified in three replicates under at least one experimental condition. Last, the iBAQ data were log_2_ transformed, and missing values were imputed from a normal distribution (width = 0.3, down shift = 1.8). Differential proteins between triplicates were calculated using a fold change cutoff that was set based on the interaction background detected in WT control group, and FDR < 0.05 (Student’s *t* test with multiple corrections) (*65*).

### Statistics and reproducibility

R version 3.5.1 on Ubuntu 16.04.5 LTS was used for statistical analyses. Error bars, *P* values, and statistical tests are reported in the figure legends. BioVenn (*66*) was used to generate the Venn diagram for overlap between RNA and ChIP-seq data (www.biovenn.nl/). Statistical tests include paired or unpaired two-tailed Student’s *t* test, Fisher’s exact test, Wilcoxon rank sum test, “*N* − 1” chi-square test, Pearson correlation, and Wald test. All experiments were performed independently at least two times unless otherwise indicated.
