# Supplementary material for: Spic regulates one-carbon metabolism and histone methylation in ground-state pluripotency
Source: Sci Adv. 2023 Aug 18;9(33):eadg7997. doi: 10.1126/sciadv.adg7997 (PMC11801372; doi:10.1126/sciadv.adg7997)
Supplement: Supplementary file 1 — Figs. S1 to S5 Table S1 Legends for data S1 to S4 [file sciadv.adg7997_sm.pdf]

Science Advances
AAAS

Supplementary Materials for

Spic regulates one-carbon metabolism and histone methylation in ground-state pluripotency

Fatemeh Mirzadeh Azad *et al.*

Corresponding author: Yaser Atlasi, y.atlasi@qub.ac.uk

Sci. Adv. **9**, eadg7997 (2023)
DOI: 10.1126/sciadv.adg7997

The PDF file includes:

Figs. S1 to S5
Table S1
Legends for data S1 to S4

Other Supplementary Material for this manuscript includes the following:

Data S1 to S4

Supplementary Figure 1

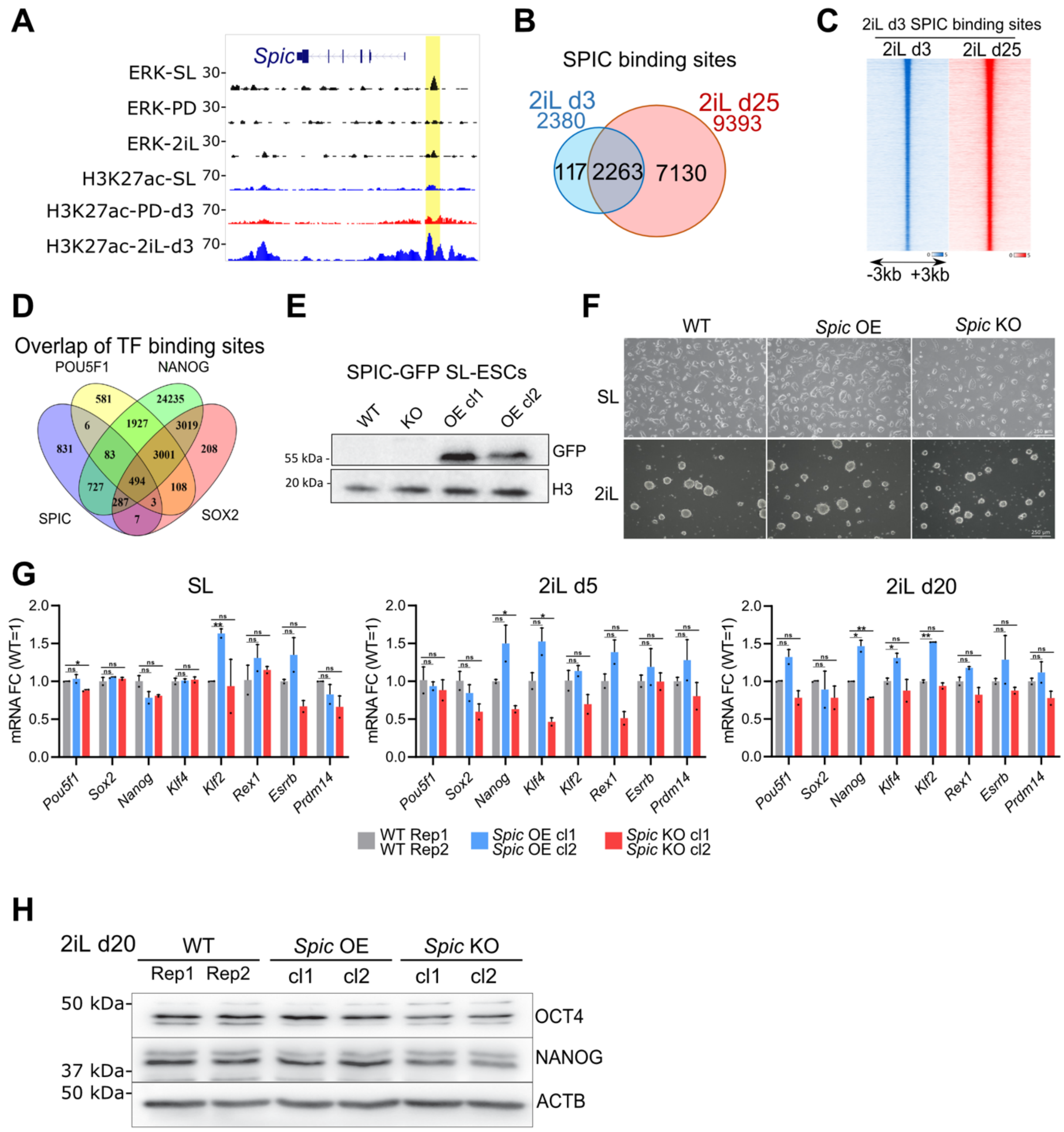

Figure S1:
(A) Genome browser view showing ERK binding at *Spic* promoter in SL but not in 2iL- or PD-cultured ESCs. **(B)** Venn diagram showing the overlap of SPIC binding sites in 2iLd3 and 2iLd25 ESCs. **(C)** Heatmap showing the SPIC binding sites detected in 2iLd3 are maintained in 2iLd25 ESCs. **(D)** Venn diagram showing that SPIC peaks overlap OCT4, NANOG, and SOX2 binding sites. Co-occupancy was assessed based on ChIP-seq data in 2iL-ESCs. **(E)** Immunoblot showing expression of SPIC-GFP in *Spic*-OE ESCs. **(F)** Microscopy pictures showing the morphology of *Spic*-KO and -OE ESCs cultured in SL or 2iL media. **(G)** qRT-PCR analysis showing expression of representative pluripotency markers in *Spic*-KO, -OE and -WT ESCs cultured in SL, 2iLd5, or 2iLd20. The bars represent the means of $n = 2$ independent clones of *Spic*-KO or -OE and $n = 2$ biological replicates of WT-ESCs. The error bars show the s.e.m and the asterisks represent P-Val < 0.05 unpaired two-tailed Student's *t*-test. **(H)** Immunoblot showing expression of the pluripotency markers OCT4 and NANOG in *Spic*-KO, -OE and -WT ESCs cultured in long-term 2iL.

Supplementary Figure 2

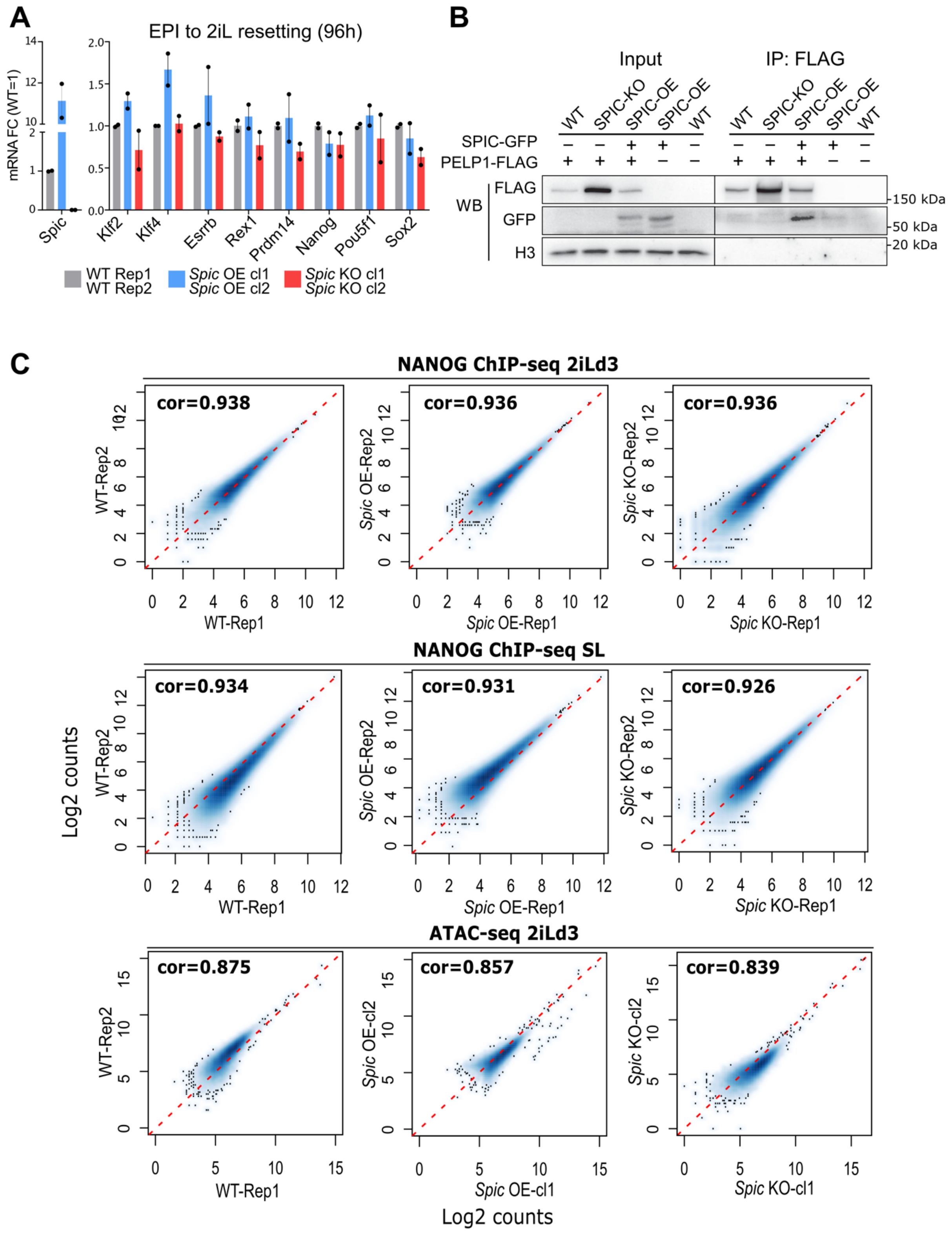

Figure S2:

(A) qRT-PCR analysis showing expression of pluripotency markers in *Spic*-KO, -OE and -WT ESCs during EpLSC to-2iL culture resetting. The bars represent the means of *n* = 2 independent clones of *Spic*-KO or -

OE and $n = 2$ biological replicates of WT-ESCs. The error bars show the s.e.m. **(B)** Immunoblot showing PELP1-SPIC interaction in 2iLd3 ESCs. ESCs stably expressing SPIC-GFP and FLAG-PELP1 were used in FLAG-PELP1 IP followed by immunoblotting for SPIC-GFP. WT ESCs and SPIC-GFP without FLAG-PELP1 were used as controls. **(C)** Scatter plots showing the correlation between individual replicates in ChIP-seq or ATAC-seq libraries. cor-values represent Pearson correlation coefficient. n=23,401 peaks for NANOG ChIP-seq in 2iLd3, n=36,969 peaks for NANOG ChIP-seq in SL, and n=20,950 peaks for ATAC-seq peaks.

Supplementary Figure 3

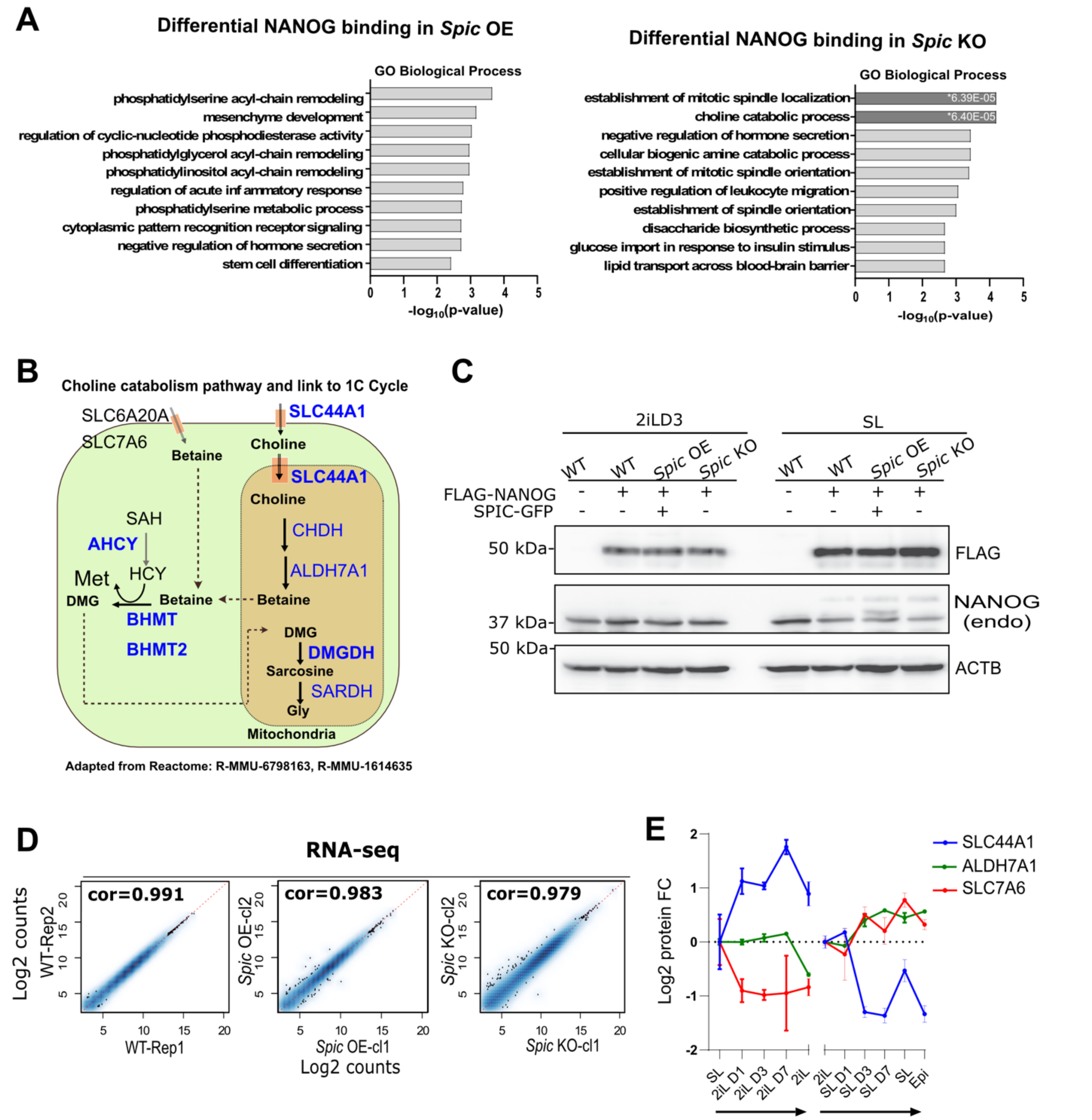

Figure S3:

(A) GO-term enrichment analysis for genes located in the vicinity of NANOG binding sites that are sensitive to *Spic* overexpression (n=528 genes) or *Spic* depletion (n=299 genes). Only NANOG binding sites with overlapping SPIC peaks are used in this analysis. **(B)** Schematic representation of genes involved in choline metabolism. Genes highlighted in bold, and blue represent genes in vicinity to NANOG-SPIC binding sites that show less NANOG binding in *Spic* KO cells. **(C)** Immunoblot showing comparable level of NANOG expression (exogenous FLAG-NANOG and endogenous NANOG) in *Spic*-KO, -OE, and -WT ESCs cultured in SL or 2iLd3. **(D)** Scatter plots showing the correlation between individuals replicates in RNA-seq libraries generated in 2iLd1 ESCs. cor-values represent Pearson correlation coefficient. n=14,398 genes. **(E)** Graph showing the expression of proteins involved in choline metabolism (SLC44A1, ALDH7A1) or betaine uptake (SLC7A6) during SL-2iL ESC transitions.

Supplementary Figure 4

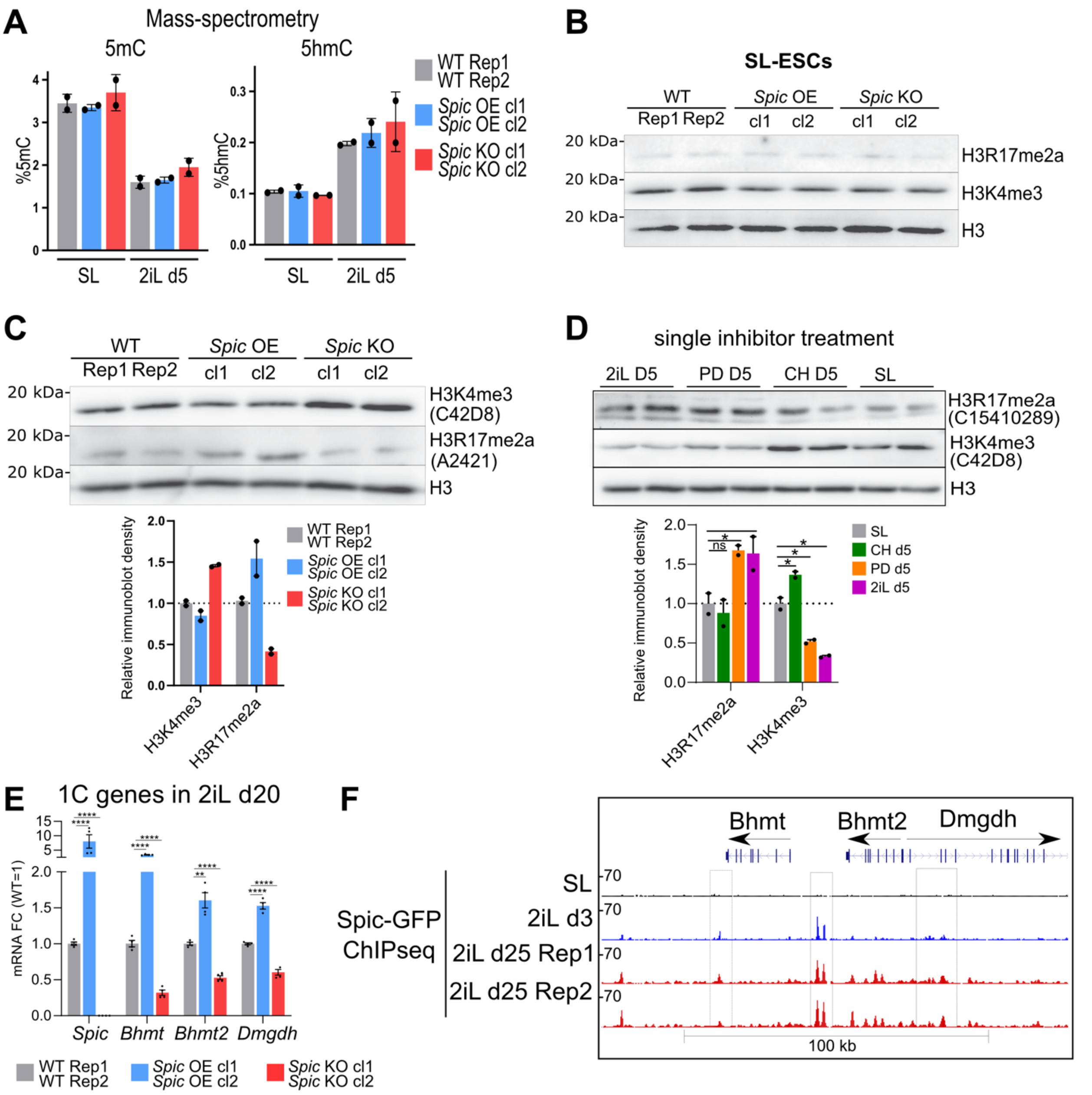

Figure S4:

(A) Graph showing the levels of 5mC and 5hmC as measured by mass-spectrometry in SL- and 2iLd5-ESCs. The bars represent the means of n = 2 independent clones of *Spic*-KO or -OE and n = 2 biological replicates of WT-ESCs. **(B)** Immunoblot showing similar levels of H3K4me3 and H3R17me2a in *Spic*-KO, OE, and -WT ESCs cultured in SL medium. Two independent clones of *Spic*-KO or -OE and n = 2 biological replicates of WT-ESCs are used. **(C)** Immunoblot showing levels of H3K4me3 and H3R17me2a in *Spic*-KO, OE, and -WT ESCs cultured in 2iLd5. An independent antibody for each mark was used when compared to **Fig 5F**. Bar plot represents the densitometry quantification of the immunoblot. Two independent clones of *Spic*-KO or -OE and n = 2 biological replicates of WT-ESCs are used. **(D)** Immunoblot showing the levels of H3K4me3 and H3R17me2a WT-ESCs cultured for 5 days in single inhibitor (PD0325901/LIF or CHIR99021/LIF) or 2iL media. An independent antibody for each mark was used when compared to **Fig 5G**. Bar plot represents the densitometry quantification of the immunoblot. n = 2 biological replicates.

(E) qRT-PCR showing expression of 1C metabolism genes in *Spic*-KO, -OE and -WT ESCs cultured in long-term 2iL. The bars represent the means of *n* = 2 independent clones of *Spic*-KO or -OE and *n* = 2 biological replicates of WT-ESCs. **(F)** Genome browser view showing SPIC binding at *Bhmt*, *Bhmt2*, and *Dmgdh* loci in SL, 2iLd3, 2iLd25 ESCs**.**

Supplementary Figure 5

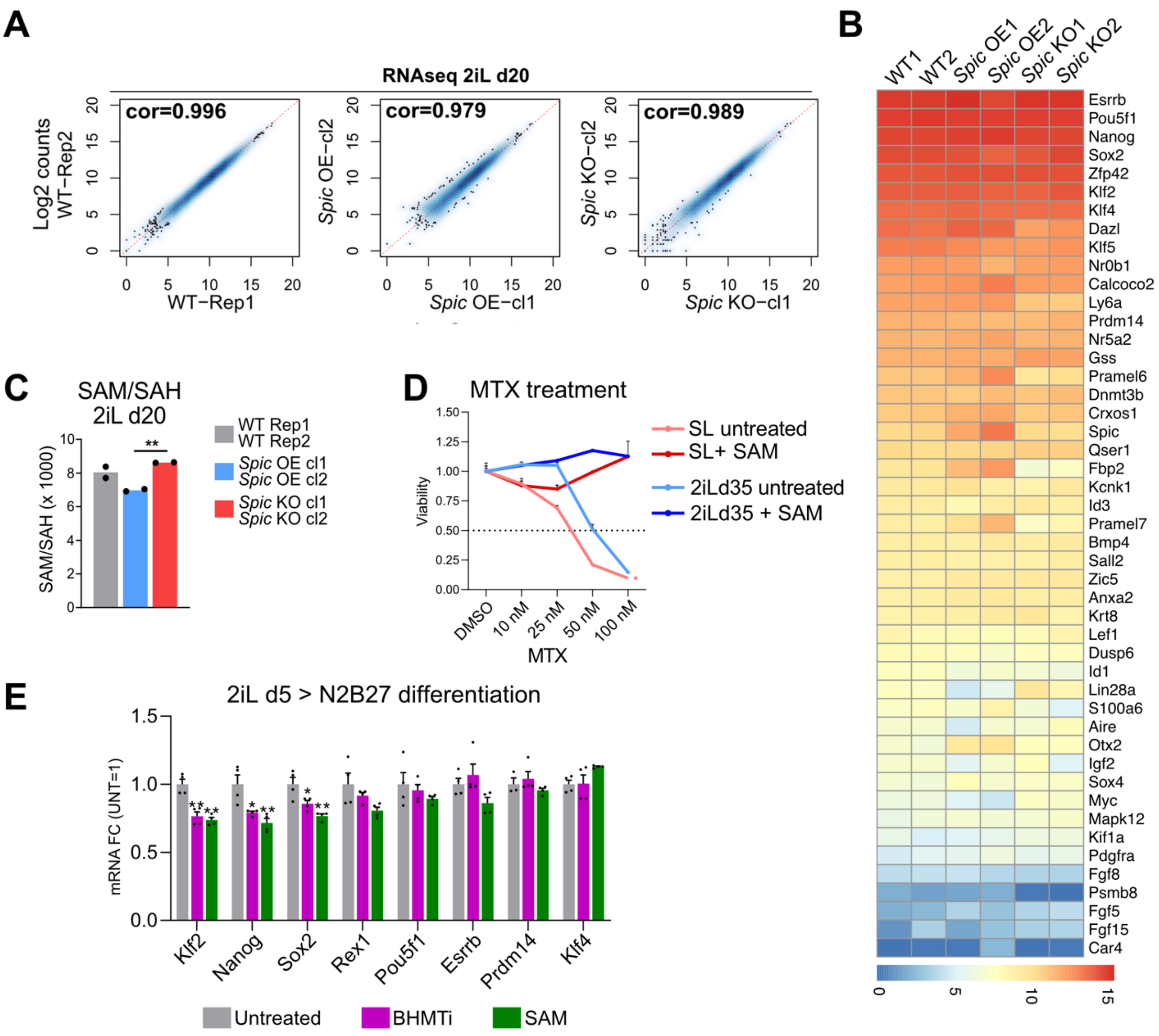

Figure S5:
(A) Scatter plots showing the correlation between individual replicates in RNA-seq libraries generated in 2iLd20 ESCs. cor-values represent Pearson correlation coefficient. n=12,871 genes. **(B)** Heatmap displaying the expression levels of select pluripotency-associated genes in RNA-seq data obtained from 2iLd20 *Spic*-KO, -OE, and -WT ESCs**. (C)** Graph showing ratio of SAM/SAH as measured by mass-spectrometry in 2iLd20-ESCs. The bars represent the means of $n = 2$ independent clones of *Spic*-KO or -OE and $n = 2$ biological replicates of WT-ESCs. **(D)** Methotrexate response in WT ESCs cultured in SL or 2iLd35. The lines represent the mean of DMSO normalized viability of n= 2 biological replicates. **(E)** qRT-PCR showing expression of representative pluripotency markers in WT-ESCs treated with SAM or BHMTi (CBHcy) for 5 days during SL-to-2iLd5 transition. The bars show the mean of n= 2 biological replicates per treatment condition. In all graphs, the error bars show the s.e.m and the asterisks represent P-Val < 0.05 unpaired two-tailed Student's *t*-test.

Table S1. **Materials**

REAGENTS	SOURCE	IDENTIFIER
Antibodies		
Anti-H3	Cell signaling	9715s
Anti-H3K4me3	Diagenode	C15410003
Anti-H3K4me3	Cell signaling	C4208
Anti-H3K4me2	Millipore	07-030
Anti-H3K4me1	Diagenode	Bp140
Anti-H3K27me3	Diagenode	C15410195
Anti-H3K36me3	Diagenode	Pab-192-050
Anti-H3K9me3	Diagenode	C15410193
Anri-H3R17me2a	Diagenode	C15410289
Anti-H3R17me2a	Abclonal	Wh284668
Anti-Flag M2	Sigma	F3165
Anti-GFP	Abcam	Ab290
Anti-BHMT	Proteintech	15965-1-ap
Anti-BHMT	Abcam	Ab96415
Anti-BHMT2	Proteintech	16351-1-1p
Anti-DMGDH	Proteintech	24813-1-ap
Anti-NANOG	Proteintech	4295-1-AP
Anti-OCT4	Proteintech	11263-1-AP
Anti-ACTB	Sigma	A1978
Anti-GAPDH	Abcam	Ab9485
Anti-Rabbit -HRP	Dako	P0217
Anti-Mouse-HRP	Dako	P0260
Bacterial and virus strains		
DH10B competent cells	Invitrogen	18297-010
Chemicals, peptides, and recombinant proteins		
CHIR99021-CT99021	Axon	1386
PD0325901	Axon	1408
LIF	Millipore	Esg1107
FBS	Gibco	10270-106
NEAA	Gibco	11140-035
Na pyruvate	Gibco	11360-070
Glutamax	Gibco	35050-038
Penicillin/Streptomycin	Gibco	15140122
KnockOut Serum Replacement	Gibco	10828028
bFGF	Peprotech	100-18B
Activin A	Peprotech	120-14E
Fibronectin	Millipore	Fc010
Puromycin	Gibco	a11138-03
Geneticin	Gibco	10131-035
DMEM	Gibco	41965-039
NDiff 227	TaKaRa	Y40002
Ligase	Invitrogen	IVGN2104
Bbsi	Thermo Scientific	ER1011
AgeI	NEB	R3552
NotI	NEB	R3189
DNA Degradase Plus	Zymo Research	E2020

Phusion Plus DNA polymerase	Thermo Scientific	F630L
Dynabeads protein A	Invitrogen	10001D
Dynabeads protein G	Invitrogen	10004D
Proteinase k	Invitrogen	25530049
Digitonin	Promega	G9441
ChromoTek GFP-Trap® Agarose	Proteintech	GTA-200
ANTI-FLAG® M2 Affinity Gel	Sigma	A2220
Benzonase	Merk	E1014
cOmplete™ Protease Inhibitor Cocktail	Sigma	11836145001
CBHcy (S-(4-Carboxybutyl)-D,L-homocysteine; 5-(3-Amino-3-carboxypropyl) sulfanyl-pentanoic acid)	AOBIOUS	AOB2142
Methotrexate	Sigma	454126
S-(5'-Adenosyl)-L-methionine iodide	Sigma	A4377
Critical commercial assays		
iTaq™ Universal SYBR® Green Supermix	Biorad	1725124
MinElute PCR Purification Kit	Qiagen	28004
NEBNext High-Fiedility 2X PCR Master Mix	NEB	M0541L
Lipofectamine 3000	Invitrogen	L3000001
KAPA Hyperprep Kit	Roche	KK8504
KAPA RNA HyperPrep Kit with RiboErase (HMR)	Roche	KK8560
Superscript III Reverse transcriptase	Invitrogen	18080044
Tagment DNA Enzyme and Buffer kit	Illumina	20034197
RNeasy mini kit	Qiagen	74106
SuperSignal™ West Pico PLUS Chemiluminescent Substrate	Invitrogen	34579
Pierce™ BCA Protein Assay Kit	Invitrogen	23225
Wizard® Genomic DNA Purification Kit	Promega	A1120
Alkaline Phosphatase Detection Kit	Sigma	SCR004
CellTiter-Glo®	Promega	G7570
Experimental models: Cell lines		
$E14^{(SpicGFP)}$	This paper	NA
$E14^{(SpicGFP, NanogFLAG)}$	This paper	NA
$E14^{(Spic-/-, NanogFLAG)}$	This paper	NA
$E14^{(NanogFLAG)}$	This paper	NA
$E14^{(SpicGFP, Pelp1FLAG)}$	This paper	NA
$E14^{(Spic-/-, Pelp1FLAG)}$	This paper	NA
$E14^{(Pelp1FLAG)}$	This paper	NA
$E14^{(Spic-/-)}$	This paper	NA
Oligonucleotides		
qPCR Primer: Pou5F1-F: TTGAGAACCGTGTGAGGTGG	This paper	NA
qPCR Primer: Pou5F1-R: TCGGGCACTTCAGAAACATG	This paper	NA
qPCR Primer: Nanog-F: AGCAGAAGATGCGGACTGTGTT	This paper	NA
qPCR Primer: Nanog-R CCTTGAGTGCACACAGCTGG	This paper	NA
qPCR Primer: Sox2-F: GACCGTTTTCGTGGTCTTGT	This paper	NA
qPCR Primer: Sox2-R: ACGATATCAACCTGCATGGAC	This paper	NA
qPCR Primer: Rex1-F: AGGCCAGTCCAGAATACCAG	This paper	NA
qPCR Primer: Rex1-R: TAGGTATCCGTCAGGGAAGC	This paper	NA
qPCR Primer: Prdm14-F: CAGCCAAGCAATTTGCACTA	This paper	NA
qPCR Primer: Prdm14-R: ACCTGGCATTTTCATTGCTC	This paper	NA
qPCR Primer: Esrrb-F:CGTCCTCGGGCATTGATG	This paper	NA

qPCR Primer: Esrrb-R:CTCGTAGCTCTTGCGGCA	This paper	NA
qPCR Primer: Klf4-F:AGACCAGATGCAGTCACAAGTC	This paper	NA
qPCR Primer: Klf4-R:CCCAGTCACAGTGGTAAGGTTT	This paper	NA
qPCR Primer: Klf2-F:ACCAAGAGCTCGCACCTAAA	This paper	NA
qPCR Primer: Klf2-R: GTGGCACTGAAAGGGTCTGT	This paper	NA
qPCR Primer: Spic-F:AGAGGTGTAACAAATGGTTCTG	This paper	NA
qPCR Primer: Spic-R:CTGGCTTTCAGCTATGTTCTG	This paper	NA
qPCR Primer: Bhmt-F:ATGGCAGCTGGGGAAGTGGT	This paper	NA
qPCR Primer: Bhmt-R:AGTGGCCTCCTTCTGCTGCA	This paper	NA
qPCR Primer:Bhmt2-F:CCGGTTTGGGCCCTGGACTA	This paper	NA
qPCR Primer: Bhmt2-R:ACCCTCCCTTGCCACAGTCC	This paper	NA
qPCR Primer:Dmgdh-F:CAATGTCGTCAATGGCCCTAT	This paper	NA
qPCR Primer:Dmgdh-R: GAACTTGCCTACCCCACCAG	This paper	NA
qPCR Primer: Actb-F: AGTGTGACGTTGACATCCGT	This paper	NA
qPCR Primer:Actb-R: TGCTAGGAGCCAGAGCAGTA	This paper	NA
qPCR Primer: Gapdh-F:TTCACCACCATGGAGAAGGC	This paper	NA
qPCR Primer: Gapdh-R:CCCTTTTGGCTCCACCCT	This paper	NA
gRNA Oligo for CRISPR mediated KO: Spic-gRNA1-sense: CACCGAACCCTTATCCTCACGTCAG	This paper	NA
gRNA Oligo for CRISPR mediated KO: Spic-gRNA1-antisense: AAACCTGACGTGAGGATAAGGGTTC	This paper	NA
gRNA Oligo for CRISPR mediated KO: Spic-gRNA2-sense: CACCGCAGCTATGTTCTGTACGGAT	This paper	NA
gRNA Oligo for CRISPR mediated KO: Spic-gRNA2-antisense: AAACATCCGTACAGAACATAGCTGC	This paper	NA
PCR Primer: Spic-edit-check-F: CCGTAGAGGGAATGGGTTATG	This paper	NA
PCR Primer: Spic-edit-check-R: CAGGATTGCAGGTTTGAAGAC	This paper	NA
PCR Primer for 3XFlag-Pelp1 construct: Pelp1-CDS-F: ATGGCGGCGGCCGTTCTTAG	This paper	NA
PCR Primer for 3XFlag-Pelp1 construct: NotI-Pelp1-CDS-R: GAGCGGCCGCCTACGAGTCAGGCTCTGTAGCAGGTG	This paper	NA
PCR Oligo for 3XFlag-Pelp1 construct: 3X Flag sense: ACCGGTAATGGACTACAAAGACCATGACGGTGATTATAAA GATCATGACATCGATTACAAGGATGACG	This paper	NA
PCR Oligo for 3XFlag-Pelp1 construct: 3X Flag antisense: GCGGCCGCACGCGTCTCGAGCCCCGGGGCTAGCGAGCTC ACCCTTGTCATCGTCATCCTTGTAATCGATGTC	This paper	NA
PCR Primer for 3XFlag-Pelp1 construct: AgeI-3XFlag-F: ACCGGTAATGGACTACAAAGACCATGAC	This paper	NA
PCR Primer for 3XFlag-Pelp1 construct: 3XFlag-R: GCCCGCAGAGGCCCCACTAAG	This paper	NA
Recombinant DNA		
Plasmid: pPYCAG-Flag-Nanog-3Gly-PIP	Gagliardi et al (*49*)	NA
Plasmid: pSpCas9(BB)-2A-Puro	Addgene	62988
Bac clone: SPIC-GFP	This paper	NA
Plasmid: pPy-CAG-Cre::ERT2-IRES-BSD	Addgene	48760
Software and algorithms		

Bowtie2	Langmead et al (*53*)	http://bowtie-bio.sourceforge.net/bowtie2/index.shtml
STAR	Dobin et al (*50*)	https://github.com/alexdobin/STAR
Samtools	Li et al (*54*)	http://www.htslib.org/
Bedtools	Quinlan et al (*55*)	https://bedtools.readthedocs.io/en/latest/
Deeptools	Ramirez et al (*56*)	https://deeptools.readthedocs.io/en/develop/content/installation.html
HTseq	Anders et al (*51*)	https://htseq.readthedocs.io/en/master/
Picard		https://broadinstitute.github.io/picard/
Macs2	Zhang et al (*57*)	https://pypi.org/project/MACS2/
BedGraphtoBigWig		https://www.encodeproject.org/software/bedgraphtobigwig/
R		https://cran.r-project.org/
DESeq2	Love et al (*52*)	http://bioconductor.org/packages/release/bioc/html/DESeq.html
EnrichR	Chen et al (*60*)	https://maayanlab.cloud/Enrichr/
Fluff	Georgiou et al (*58*)	https://fluff.readthedocs.io/en/latest/index.html
Perseus	Tyanova et al (*64*)	https://maxquant.net/perseus/
Biovenn	Hulsen et al (*66*)	http://www.biovenn.nl/

Data S1:
List of all next generation sequencing samples generated in this study

Data S2:
Table summarizing IP-MS data

Data S3:
Table summarizing ChIP-seq data

Data S4:
Table summarizing RNA-seq data
